# Automatic Recognition of Macaque Facial Expressions for Detection of Affective States

**DOI:** 10.1523/ENEURO.0117-21.2021

**Published:** 2021-12-09

**Authors:** Anna Morozov, Lisa A. Parr, Katalin Gothard, Rony Paz, Raviv Pryluk

**Affiliations:** 1 Department of Neurobiology, Weizmann Institute of Science, Rehovot 7610001, Israel; 2 Yerkes National Primate Research Center, Emory University, Atlanta, Georgia 30329; 3 Department of Psychiatry and Behavioral Science, Emory University, Atlanta, Georgia 30322; 4 Department of Physiology, College of Medicine, University of Arizona, Tucson, Arizona 85724

## Abstract

Internal affective states produce external manifestations such as facial expressions. In humans, the Facial Action Coding System (FACS) is widely used to objectively quantify the elemental facial action units (AUs) that build complex facial expressions. A similar system has been developed for macaque monkeys—the Macaque FACS (MaqFACS); yet, unlike the human counterpart, which is already partially replaced by automatic algorithms, this system still requires labor-intensive coding. Here, we developed and implemented the first prototype for automatic MaqFACS coding. We applied the approach to the analysis of behavioral and neural data recorded from freely interacting macaque monkeys. The method achieved high performance in the recognition of six dominant AUs, generalizing between conspecific individuals (*Macaca mulatta*) and even between species (*Macaca fascicularis*). The study lays the foundation for fully automated detection of facial expressions in animals, which is crucial for investigating the neural substrates of social and affective states.

## Significance Statement

MaqFACS is a comprehensive coding system designed to objectively classify facial expressions based on elemental facial movements designated as actions units (AUs). It allows the comparison of facial expressions across individuals of same or different species based on manual scoring of videos, a labor- and time-consuming process. We implemented the first automatic prototype for AUs coding in macaques. Using machine learning, we trained the algorithm on video frames with AU labels and showed that, after parameter tuning, it classified six AUs in new individuals. Our method demonstrates concurrent validity with manual MaqFACS coding and supports the usage of automated MaqFACS. Such automatic coding is useful not only for social and affective neuroscience research but also for monitoring animal health and welfare.

## Introduction

Facial expressions are both a means of social communication and also a window to the internal states of an individual. The expression of emotions in humans and animals was discussed first by Darwin (1872) in his eponymous treatise in which he attributed the shared features of emotional expression in multiple species to a common ancestor. Further elaboration of these ideas came from detailed understanding of the neuromuscular substrate of facial expressions (i.e., the role of each muscle in moving facial features into configurations that have social communicative value). These studies brought to light the homologies, but also the differences in how single facial muscles, or groups of muscles give rise to a relatively stereotypical repertoire of facial expressions (Ekman, 1989; Ekman and Keltner, 1997; Burrows et al., 2006; Vick et al., 2007; Parr et al., 2010).

The affective states that give rise to facial expressions are instantiated by distinct patterns of neural activity (Panksepp, 2004) in areas of the brain that have projections to the facial motor nucleus in the pons. The axons of the motor neurons in the facial nucleus distribute to the facial musculature, including the muscles that move the pinna (Jenny and Saper, 1987; Welt and Abbs, 1990). Of all possible facial muscle movements, only a small set of coordinated movements give rise to unique facial configurations that correspond, with some variations, to primary affective states. Human studies of facial expressions proposed six primary affective states or “universal emotions” that were present in facial displays across cultures (Ekman and Friesen, 1986; Ekman and Oster, 1979; Ekman and Friesen, 1988; for review, see Ekman et al., 2013). The cross-cultural features of facial expressions allowed the development of an anatomically based Facial Action Coding System (FACS; Friesen and Ekman, 1978; Ekman et al., 2002). In this system, a numerical code is assigned for each elemental facial action that is identified as an action unit (AU). Considering the phylogenetic continuity in the facial musculature across primate species (Burrows and Smith, 2003; Burrows et al., 2006, 2009; Parr et al., 2010), a natural extension of human FACS was the homologous Macaque FACS (MaqFACS; Parr et al., 2010), developed for coding the facial action units in Rhesus macaques (for multispecies FACS review, see Waller et al., 2020).

The manual scoring of AUs requires lengthy training and a meticulous certification process for FACS coders, which is a time-consuming process. Therefore, considerable effort has been made toward the development of automatic measurement of human facial behavior (Sariyanidi et al., 2015; for review, see Barrett et al., 2019). These advances do not translate seamlessly to macaque monkeys, and, importantly, similar developments are desirable because macaques are commonly used to investigate and understand the neural underpinnings of communication via facial expressions (Livneh et al., 2012; Pryluk et al., 2020). We therefore aimed to develop and test an automatic system to classify AUs in macaques, one that would allow comparison of elicited facial expressions and neural responses at similar temporal resolutions.

Like humans, macaque monkeys do not normally activate a full set of action units required for a classical stereotypical expression, and partial sets of uncommon combination of action units are also probable and give rise to mixed or ambiguous facial expressions (Chevalier-Skolnikoff, 1973; Ekman and Friesen, 1976). Therefore, we chose to classify not only the fully developed facial expressions (Blumrosen et al., 2017) but also action units that were shown to play a role in the exhibition of affective states and social communication among macaque monkeys. We included even relatively rare facial expressions as long as certain action units were reliably involved in these expressions. We test the automatic recognition of facial configurations and show that it generalizes to new situations, between conspecific individuals, and even across macaque species. Together, this work demonstrates concurrent validity with manual MaqFACS coding and supports the usage of automated MaqFACS in social and affective neuroscience research, as well as in monitoring animal health and welfare.

## Materials and Methods

### Video datasets

We used videos from two different datasets. The first, the Rhesus dataset (RD), consists of 53 videos from 5 Rhesus macaques (selected from 10 Rhesus monkeys). Part of this dataset was used for training and testing our system within and across Rhesus subjects. The second, the Fascicularis dataset (FD), includes two videos from two Fascicularis macaques and was used only for testing our system across Fascicularis subjects.

All the videos in both sets capture frontal (or near-frontal) views of head-fixed monkeys. The video-frames were coded for the AUs present in each frame (none, one, or many).

The subjects and the videos for RD were selected with respect to the available data in FD, considering the scale similarity, the filming angle and the AU frequencies occurring in the videos.

### The Rhesus macaque facial action coding system

**Figure 1.**
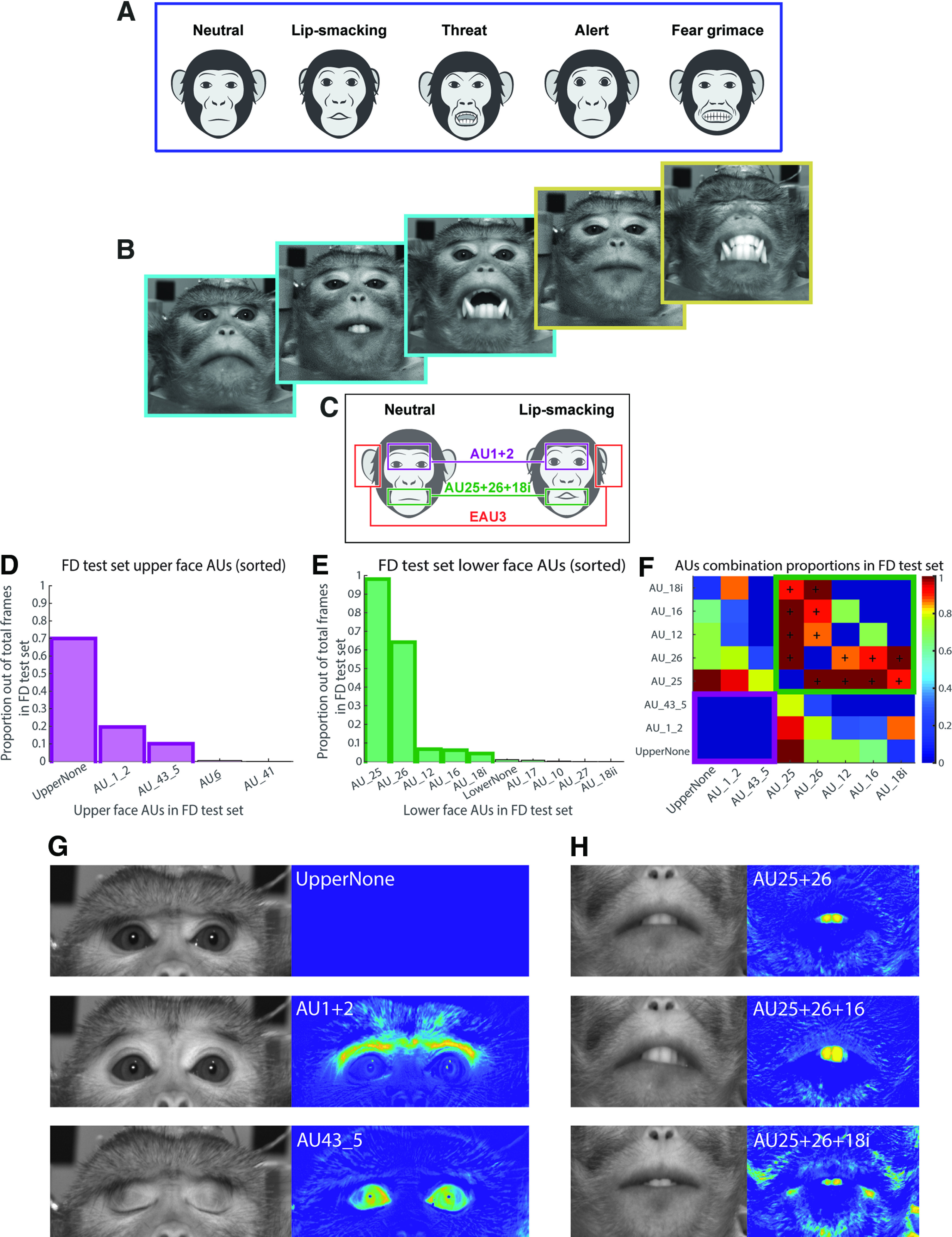
Motivation for using automatic MaqFACS to analyze facial expressions. ***A***, The stereotypical facial expressions in macaque monkeys include the “neutral,” “lip-smacking,” “threat,” “alert,” and “fear grimace” expressions (Altmann, 1962; Hinde and Rowell, 1962). ***B***, Some of the facial expressions that monkeys produce during the experiments that require head immobilization match the stereotypical expressions produced during natural behaviors (e.g., the three images with blue frames on the left correspond to the neutral, lip-smacking, and threat expressions). We have also observed facial expressions that were less frequently described in the literature (two images with yellow frames on the right). ***C***, A comparison between the neutral and lip-smacking facial expression shows that the lip-smacking example contains AU1 + 2 (Brow Raiser) in the upper face, AU25 + 26 + 18i (Lips part, Jaw drop, and True Pucker) in the lower face, and EAU3 (Ear Flattener) in the ear region. ***D***, The proportion of each upper face AU in the FD test set. Bars with the solid outline (first three highest bars) represent the most frequent AUs, which were chosen for the analysis in this work. UpperNone - no coded action in the upper face, AU1+2 - brow raiser, AU43_5 - eye closure, AU6 - cheek raiser, AU41- glabella lowerer. ***E***, Same as ***D***, but for lower face. First five most frequent AUs were chosen for the analysis. ***F***, Proportion matrix of AU combinations in the FD test set, for the most frequent AUs. Cells inside the magenta (bottom left) and green frames (top right) represent the combinations of upper face and lower face AUs, correspondingly. AUs that frequently occurred in combination with other AUs (in the upper face or the lower face, separately) are denoted by “+.” Cell values were calculated as the ratio between the number of frames containing the combination of the two AUs and the total number of frames containing the less frequent AU. ***G***, Left, Images of upper face AUs from the FD test set. UpperNone, No coded action in the upper face; AU1 + 2, Brow Raiser; AU43_5, Eye closure. Right, The difference of the images from the neutral face image. ***H***, Same as ***G***, but for lower face. AU25 + 26, Lips part and Jaw drop; AU25 + 26 + 16, Lips part, Jaw drop, and Lower lip depressor; AU25 + 26 + 18i, Lips part, Jaw drop, and True Pucker.

There are several stereotypical facial expressions that macaques produce (Fig. 1*A*), that represent, as in humans, only a subset of the full repertoire of all the possible facial movements. For example, Figure 1*B* represents three common facial expressions from the FD (Fig. 1*B*, left, blue) and two other facial configurations that, among others, occurred in our experiments (Fig. 1*B*, right, yellow). Therefore, to allow the potential identification of all the possible facial movements (both the common and the less common ones), we chose to work in the MaqFACS domain and to recognize AUs, rather than searching for predefined stereotypical facial expressions. The MaqFACS contains the following three main groups of AUs based on facial sectors: upper face, lower face, and ears (Parr et al., 2010). Each facial expression is instantiated by a select combination of AUs (Fig. 1*C*).

### AU selection

The criteria for AU selection for the analysis in this work were their frequencies (which should be sufficient for training and testing purposes) and the importance of each AU for affective communication ( Fig. 1*D*,*E*; Parr et al., 2010; Ballesta et al., 2016; Mosher et al., 2016). Frequent combinations of lower face AUs together with upper face AUs (Fig. 1*F*, outside the magenta and green frames) may hint at the most recurring facial expressions in the test set. For example, the UpperNone AU together with the lower face AU25 generate a near-neutral facial expression. Considering that our aim is to recognize single AUs (as opposed to complete predefined facial expressions), lower face and upper face AUs were not merged into single analysis units. This approach is also supported by the MaqFACS coding process, which is performed separately for the lower and upper faces.

The most frequent upper face AUs in the FD were the none-action AU (defined here as “UpperNone”), the Brow Raiser AU1 + 2 and AU43_5, which is a union of Eye Closure AU43 and Blink AU45 (Fig. 1*D*). The two latter AUs differ only in the movement duration, and hence were joined.

There were five relatively frequent AUs in the lower face test set (Fig. 1*E*) that we merged into several AU groupings. All AUs that mostly co-occurred with other ones (within the same face region) were analyzed as a combination rather than as single units (Fig. 1*F*, inside the green frame). The upper face AUs, however, rarely appeared as combination (Fig. 1*F*, inside the magenta frame).

Overall, our system was trained to classify the following six units: AU1 + 2, AU43_5, and UpperNone in the upper face, and AU25 + 26, AU25 + 26 + 16, and AU25 + 26 + 18i in the lower face (Fig. 1*G*,*H*, left). Although AU12 was one of the most prevalent AUs in the FD test set and often occurred in combination with other lower face AUs, it was eliminated from further analysis because it appeared too infrequently in the RD.

### Animals and procedures

All surgical and experimental procedures were approved and conducted in accordance with the regulations of the Institute Animal Care and Use Committee, following National Institutes of Health regulations and with AAALAC accreditation.

Two male Fascicularis monkeys (*Macaca fascicularis*) and 10 Rhesus monkeys (*Macaca mulatta*) were videotaped while producing spontaneous facial movements. All monkeys were seated and head fixed in a well lit room during the experimental sessions.

**Figure 2.**
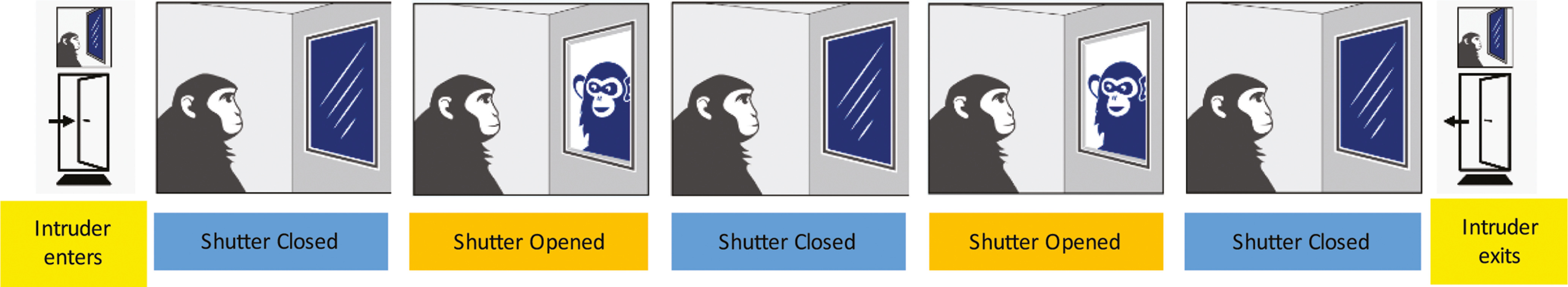
Monkey–intruder behavioral paradigm. Monkey–intruder block, The subject monkey is sitting behind a closed shutter. The intruder monkey is brought into the room and seated behind the shutter, which remains closed. The shutter opens and closes 18 times, and the monkeys are able to see each other while it is open. The subject monkey can not see any part of the intruder unless the shutter is open. At the end of the block, the shutter closes and the intruder monkey is taken out from the room (Extended Data Figs. 2-1, 2-2, 2-3, examples of monkey interactions).

The two monkeys produced facial behaviors in the context described in detail in the study by Pryluk et al. (2020; Fig. 2, Extended Data Figs. 2-1, 2-2, 2-3). The facial movements obtained during neural recordings have not been previously analyzed in terms of action units. Earlier experiments showed that self-executed facial movements recruit cells in the amygdala (Livneh et al., 2012; Mosher et al., 2016) and the anterior cingulate cortex (ACC; Livneh et al., 2012), and that neural activity in these regions is temporally locked to different socially meaningful, communicative facial movements (Livneh et al., 2012). The video data from these monkeys was captured using two cameras (model MQ013RG, Ximea; one camera for the whole face and one dedicated to the eyes), with lenses mounted on them: 16 mm (model LM16JC10M, Kowa Optical Products Co. Ltd.) for the face camera and 25 mm (model LM25JC5M2, Kowa Optical Products Co. Ltd.) for the eye camera. The frame rates of the face and eye videos are 34 frames/s (∼29 ms) and 17 frames/s (∼59 ms), respectively. The size parameters are 800 × 700 pixels for the facial videos and 700 × 300 pixels for the videos of eyes. Both video types have 8 bit precision for grayscale values. The lighting in the experiment room included white LED lamps and an infrared LED light bar (MetaBright Exolight ISO-14-IRN-24, Metaphase Technologies) for face illumination.

**Figure 3.**
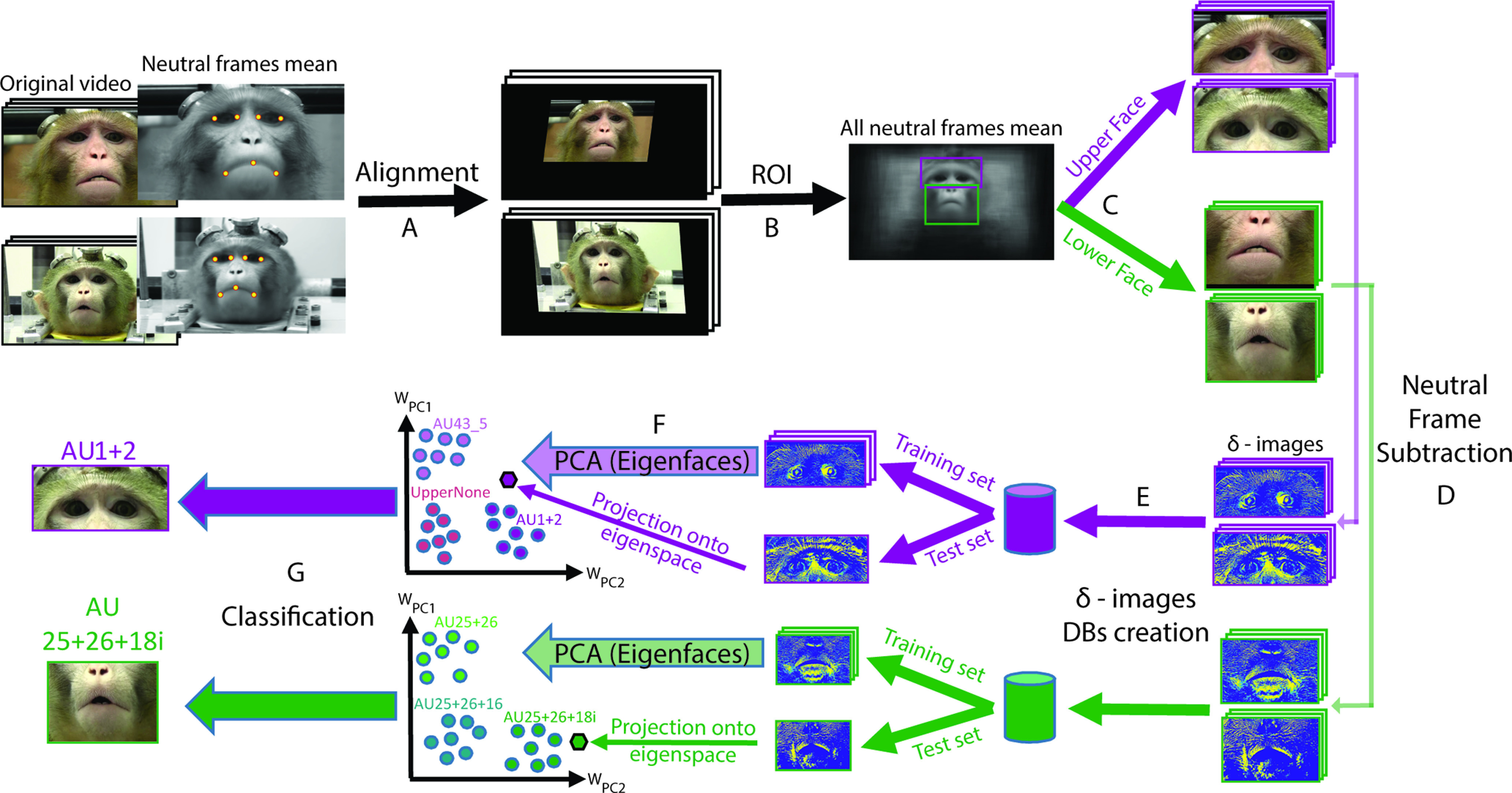
Diagram of the automatic MaqFACS AUs recognition system pipeline. ***A***, Alignment of frames from the original video stream (example of two videos from two different RD monkeys). Seven landmark points were manually selected on the mean of all neutral frames of each video. In the next step, these points were mapped to corresponding predefined positions (reference landmarks, common for all videos). The resulting affine transformation for each video was then applied to all its frames. For more examples, see Extended Data Figure 3-1. ***B***, Manual definition of upper face and lower face ROIs on the mean of all neutral frames. Magenta, Upper face ROI; green, lower face ROI. The “All neutral frames mean” image in this scheme was calculated from all RD videos. ***C***, Cropping of all the frames according to upper face and lower face ROIs. ***D***, Generation of δ-images by subtracting the optimal neutral frame of each video from all its frames. The contrast and the color map of the grayscale images were adjusted for a better representation. ***E***, Construction of lower face and upper face δ-images databases, consisting of two-dimensional matrices where each row corresponds to one image. ***F***, Eigenface extraction from the training images and projection of the training and test images onto the eigenspace (following the desired training and test sets construction). $W_{\text{PC1}}$ and $W_{\text{PC2}}$ denote the weights of PC1 and PC2, correspondingly. ***G***, Classification of the testing images to upper face and lower face AUs. KNN (and SVM) classification was applied based on the distances between the testing and the training images in the eigenspace.

10.1523/ENEURO.0117-21.2021.f2-1 Figure 2-1 Lip-smacking interactions. Examples of dynamics and progression of lip-smacking interactions captured during the monkey–intruder experiment, where the subject monkey is the first to initiate the movement. Each sequence demonstrates sample frames of the Fascicularis subject D with his head fixed (first row), along with the corresponding frames of the intruder Fascicularis monkey (second row). The subject monkey D was filmed using the facial camera (see Materials and Methods). The intruder monkey was filmed using another monitoring camera, from the direction of the subject monkey and through the opened shutter (hence, the reflections on the screen). The time presented relative to the first frame in the sequence, which starts with a neutral expression of the subject monkey. Yellow arrows indicate the change in the movement of brows, ears, and lips at the onset of the lip-smacking movement (for the subject and the intruder monkeys) and the offset of the movement (for the intruder monkey). In the example, sequence with intruder monkey B. Download Figure 2-1, TIF file.

10.1523/ENEURO.0117-21.2021.f2-2 Figure 2-2 Lip-smacking interactions. Same setup as in Figure 2-1, but with intruder monkey P. Download Figure 2-2, TIF file.

10.1523/ENEURO.0117-21.2021.f2-3 Figure 2-3 Lip-smacking interactions. Same setup as in Figure 2-1, but with intruder monkey N. Download Figure 2-3, TIF file.

10.1523/ENEURO.0117-21.2021.f3-1 Figure 3-1 Motivation for alignment. Seven reference landmark points (yellow, predefined and common for all videos) displayed on sample neutral frames of original video streams. ***A***, Sample neutral frames from five different videos of each of the five Rhesus monkeys (K, L, M, Q, R). ***B***, Sample neutral frames from one video of Rhesus monkey K. ***C***, Sample neutral frames of the two Fascicularis monkeys (D and B). Download Figure 3-1, TIF file.

The 10 Rhesus monkeys were filmed during baseline sessions as well as during provocation of facial movements by exposure to a mirror and to videos of other monkeys. Videos of facial expressions of the Rhesus macaques were recorded at 30 frames/s (∼33 ms) rate, with 1280 × 720 pixels size parameters and 24 bit precision for RGB values.

### Behavioral paradigms

The intruder task is similar to the one described in the study by Pryluk et al. (2020), including a monkey intruder instead of a human (Fig. 2, Extended Data Figs. 2-1, 2-2, 2-3). A single experimental block includes six interactions (trials) with a monkey intruder that is seated behind a fast LCD shutter (<1 ms response time, 307 × 407 mm), which is used to block the visual site. When the shutter opens, the monkeys are able to see each other. Each trial is ∼9 s, and the shutter is closed for ∼1 s between the trials. Altogether, the length of the interaction part (from the first shutter opening until its last closure) is 60 s.

We recorded the facial expressions of the subject monkey, along with monitoring the intruder monkey behavior. When the intruder monkey was brought to or out from the room (the “enter–exit” stage), the shutter was closed and the subject monkey could not see any part of the intruder unless the shutter was open. The “enter” and the “exit” phases were each 30 s long.

### Data labeling

Video data annotation was conducted using Noldus software The Observer XT (https://www.noldus.com/human-behavior-research/products/the-observer-xt). The recorded behavior coding was exported in Excel 2016 (Microsoft) format for further processing.

RD videos were labeled by an FACS-accredited (Friesen and Ekman, 1978; Ekman et al., 2002) and MaqFACS-accredited (Parr et al., 2010) coding expert. Another trained observer performed the coding of all FD videos according to the MaqFACS manual based on the study by Parr et al. (2010). Facial behavior definitions were discussed and agreed on before the coding. To ensure consistency, we checked the inter-rater reliability (IRR) for one of the two FD videos against an additional experienced coder. Our target percentage of agreement between observers was set to 80% (Baesler and Burgoon, 1987), and the IRR test resulted in 88% agreement (Extended Data Fig. 5-1).

10.1523/ENEURO.0117-21.2021.f5-1 Figure 5-1 Confusion matrix: inter-rater variability. Confusion matrix for the inter-rater variability between two experienced human coders, for a video from FD. “Other Upper” and “Other Lower” represent all the upper face and lower face labels that were not part of the task of the automatic classifier. Download Figure 5-1, DOCX file.

All the videos were coded for MaqFACS AUs along with their frequencies and intensities. Analyzed frames with no labels were considered as frames with neutral expression. Upper and lower face AUs were coded separately. This partition was inspired by observations indicating that facial actions in the lower face have little influence on facial motion in the upper face and vice versa (Friesen and Ekman, 1978). Moreover, neurologic evidence suggests that lower and upper faces are engaged differently by facial expressions and that their muscles are controlled by anatomically distinct motor areas (Morecraft et al., 2001).

### Image preprocessing

For each video from both datasets, seven landmark points (two corners of each eye, two corners of the mouth and the mouth center) were manually located on the mean image of frames with neutral expression. For image height (h) and width (w), the reference landmark points were defined by the following coordinates: 0.42 w/0.3 h and 0.48 w/0.3 h for left eye corners; 0.52 w/0.3 h and 0.58 w/0.3 h for right eye corners; 0.44 w/0.55 h for mouth left corner; 0.56 w/0.55 h for mouth right corner; and 0.5 w/0.5 h for the mouth center (Extended Data Fig. 3-1).

Affine transformations (geometric transformations that preserve lines and parallelism; e.g., rotation) were applied to all frames of all videos so that the landmark points were mapped to predefined reference locations (Fig. 3*A*, Extended Data Fig. 3-1). The alignment procedure was necessary to correct any movement, either from the alignment of the camera (angle, distance, height) or movement of the monkey, that would shift the facial landmarks between video frames. After the alignment procedure, total average image of all mean neutral expression frames was calculated. Two rectangular regions of interest (ROIs), one for the upper face and one for lower face, were marked manually on the total average image (Fig. 3*B*). Finally, all the frames were cropped according to the ROI windows (Fig. 3*C*), resulting in 396 × 177 pixel upper face images and 354 × 231 pixel lower face images. After this step, the originally RGB images were converted to grayscale. For each video, one “optimal” neutral expression frame was selected of all the neutral expression images. Difference images (δ-images) were generated by subtraction of the optimal neutral frame from all the frames of the video (Figs. 1*G*,*H*, right, 3*D*). The main idea behind this operation was to eliminate variability because of texture differences in appearance (e.g., illumination changes) and to analyze the variability of facial distortions (e.g., action units) and individual differences in facial distortion (Bartlett et al., 1996). In the last preprocessing step, upper face and lower face databases (DBs) were created by converting the δ-images to single-dimension vectors and storing them as a two-dimensional matrix containing the pixel brightness values (one dimension is the size of the total image pixels, and the second dimension represents the image quantity). The DBs were then used for the construction of training and test sets (Fig. 3*E*).

### Eigenfaces: Dimensionality reduction and feature extraction

Under controlled head-pose and imaging conditions, the statistical structure of facial expressions may be efficiently captured by features extracted from principal component analysis (PCA; Calder et al., 2001). This was demonstrated in the “EigenActions” technique (Donato et al., 1999), where the facial actions were recognized separately for upper face and lower face images (the well known “eigenfaces”). According to this technique, the PCA is used to compute a set of subspace basis vectors (referred to as the eigenfaces) for a dataset of facial images (the training set), which are then projected into the compressed subspace. Typically, only the *N* eigenvectors associated with the largest eigenvalues are used to define the subspace, where *N* is the desired subspace dimensionality (Draper et al., 2003). Each image in the training set may be represented and reconstructed by the mean image of the set and a linear combination of its principal components (PCs). The PCs are the eigenfaces, and the coefficients of the PCs in the linear combination constitute their weights. The test images are matched to the training set by projecting them onto the basis vectors and finding the nearest compressed image in the subspace (the eigenspace).

We applied the eigenfaces analysis on the training frames (the δ-images), which were first zero-meaned (Fig. 3*F*). Once the eigenvectors were calculated, they were normalized to unit length, and the vectors corresponding to the smallest eigenvalues (<10^−6^) were eliminated.

### Classification

One of the benefits of the mean subtraction and the scaling to unit vectors is that this operation projects the images into a subspace where Euclidean distance is inversely proportional to correlation between the original images. Therefore, nearest-neighbor matching in eigenspace establishes an efficient approximation to image correlation (Draper et al., 2003). Consequently, we used a K-nearest neighbors (KNN) classifier in our system. Related to the choice of classifier, previous studies show that when PCA is used, the choice of the subspace distance-measure depends on the nature of the classification task (Draper et al., 2003). Based on this notion and other observations (Bartlett et al., 1999), we chose the Euclidian distance and the cosine of the angle between feature vectors to measure similarity. In addition, to increase the generality of our approach and to validate our results, we also tested a support vector machine (SVM) classifier. To evaluate the performance of the models, we define a classification trial as successful if the AU predicted by the classifier was the same as in the probe image. To further justify the classification of AUs separately for upper face and lower face ROIs, it is worth mentioning that evidence suggests that PCA-based techniques performed on full-face images lead to poorer performance in emotion recognition compared with separate PCA for the upper and lower regions (Padgett and Cottrell, 1997; Bartlett, 2001).

To train a classification model for AU recognition, we used the weights of the PCs as predictors. To predict the AU of a new probe image, the probe should be projected onto the eigenspace to estimate its weights (Fig. 3*F*). Once the weights are known, AU classification may be applied. The output of the classifier of each facial ROI is the AU that is present in the frame (Fig. 3*G*). To increase the generality of our approach and to validate our results, we used both KNN and SVM classifiers.

### Parameter selection

In the KNN classification, we examined the variation of the following three main parameters: the number of the eigenspace dimensions (PCs); the subspace distance metric; and *k*, the number of nearest neighbors in the KNN classifier.

Multiple ranges of PCs were tested (the “pcExplVar” parameter) from PC quantity that cumulatively explains 50% of the variance of each training set to 95%, *k* was varied from 1 to 12 nearest neighbors, and the performance was also tested with Euclidian and cosine similarity measures. For each training set and parameter set, the features were recomputed and the model performance was re-estimated. The process was repeated across all the balanced training sets (see Data undersampling). The parameters of the models and the balanced training sets were selected according to the best classification performance in the validation process.

### Data undersampling

The training sets in this study were composed of RD frames from AU1 + 2, AU43_5, and UpperNone categories in the upper face, and AU25 + 26, AU25 + 26 + 16, and AU25 + 26 + 18i in the lower face (in a nonoverlapping manner relative to each ROI). For the training purposes, for both ROIs, the RD frames were randomly undersampled 3–10 times (depending on the data volume), producing the “balanced training sets.” The main reason for this procedure was to balance the frame quantity of the different AUs in the training sets (He and Garcia, 2009). For each dataset, the size of the balanced training set was defined based on the smallest category size (Table 1). As a result, for the training processes in our experiments, we used upper face and lower face balanced training sets of size 3639 and 930 frames each, correspondingly.

**Table 1:** Data undersampling (RD)

	AU1 + 2	AU43_5	UpperNone	Undersampled per class	Total balanced training set
Upper face					
frames, *n*	1213	∼19,500	∼150,000	1213	3639

	AU25 + 26 + 16	AU25 + 26 + 18i	AU25 + 26	Undersampled per class	Total balanced training set
Lower face					
frames, *n*	310	∼15,000	∼15,000	310	930

In the upper face, the smallest category was AU1 + 2 with only 1213 frames (in total, from all RD subjects). On the contrary, AU43_5 category had ∼19,500 frames (after eliminating RD AU45 frames because of time synchronization errors), and UpperNone class included >150,000 images. Consequently, balanced training sets were generated with each including all of the AU1 + 2 frames, and randomly selected 1213 frames from AU43_5 along with 1213 randomly selected UpperNone frames. Therefore, the upper face balanced training sets were each composed of 3639 frames. The same was done for the lower face, where the smallest category was AU25 + 26 + 16 with only 310 frames. Categories AU25 + 26 + 18i and AU25 + 26 contained >15,000 images each. Accordingly, each lower face balanced training set included 930 frames.

It should be noted that the undersampling procedure influences only the composition of the training sets but not of the test sets (only the frames for training are selected from the balanced training sets). The test set composition depends on the subjects and the videos selected for the testing, and considers all the available frames that fit the task criteria (consequently, they are the same across all the balanced training sets).

### Validation and model evaluation

We tested three types of generalization. For each type of generalization, the performance was evaluated independently for upper face and lower face, using holdout validation for the Fascicularis data (Geisser, 1975) and leave-one-out cross-validation (CV) for the Rhesus data (Tukey, 1958). The leave-one-out technique is advantageous for small datasets because it maximizes the available information for training, removing only a small amount of training data in each iteration. Applying the leave-one-out CV, data from all subjects (or videos) but one, were used for the system training, and the testing was performed on the one remaining subject (or video). We designed the CV partitions constraining an equal number of frames in each class of the training sets. In both the leave-one-out CV and the holdout validation, images of the test sets were not part of the corresponding training sets, and only the training frames were retrieved from the balanced training sets. To ensure the data sufficiency for training and testing, a subject (or video) was included in the partition for CV only if it had enough frames of the three AU classes (separately for upper face and lower face).

For each generalization type, the training and the testing sets were constructed as follows. (1) Within-subject (Rhesus): for each CV partition, frames from all videos but one, from the same Rhesus subject, were used for training. Frames of the remaining video were used for testing. Performed on RD, on three balanced training sets. To be included in a CV partition for testing, the training and the test sets for a video had to consist of at least 20 and 5 frames/class, correspondingly. Some subjects did not meet the condition, and this elimination process resulted with three subjects for upper face and four subjects for lower face CV. (2) Across subjects (Rhesus): for each CV partition, frames from all videos of all Rhesus monkeys but one were used for training. Each test set was composed of frames from videos of the one remaining monkey. Performed on RD, on three balanced training sets. To be included for testing in the CV, the training and the test sets for a subject had to contain at least 150 and 50 frames of each class, correspondingly. In total, four subjects were included in the upper face testing and three subjects were included in the lower face testing. (3) Across species: frames from all videos of the five Rhesus monkeys were used for training. Frames from the two Fascicularis monkeys were used for validation and testing. In this case, a holdout model validation was performed independently for each Fascicularis monkey (each subject had a different set of model parameters selected). For this matter, each Fascicularis monkey’s dataset was randomly split 100 times in a stratified manner (so the sets will have approximately the same class proportions as in the original dataset) to create two sets: a validation set with 80% of the data; and a test set with 20% of the data. Overall, the training sets were constructed from 10 balanced training sets of the Rhesus dataset. Validation and test sets (produced by 100 splits in total) included 80% and 20% of the Fascicularis dataset, correspondingly. The best model parameters were selected according to the mean performance in validation set (over 100 splits), and the final model evaluation was calculated based on the test set mean performance (over the 100 splits, as well).

### Performance measures

Although the balanced training sets and the CV partitions were constructed to maintain the total number of actions as even as possible, the subjects and their videos in these sets possessed different quantities of actions. In addition, while we constrained the sizes of the classes within each training set to be equal, we used the complete available data for the test sets. Since the overall classification correct rate (accuracy) may be an unreliable performance measure because of its dependence on the proportion of targets to nontargets (Pantic and Bartlett, 2007), we also applied a sensitivity measure (Benitez-Quiroz et al., 2017) for each AU (where the target is the particular AU and the nontargets are the two remaining AUs).

We used the average sensitivity measure [average true positive rate ( $\bar{TPR}$)] to select the best parameter set. To compare the performance of the classifiers, we present the generalization results on a subject (i.e., individual monkey) level (rather than video) for each classification type. Performance on Fascicularis dataset is reported as the mean performance of two parameter sets (one set per subject).

### Single-neuron activity analysis

We analyzed a subset of neurons that was previously reported in the study by Pryluk et al. (2020) and corresponded to the relevant blocks of monkey–monkey interactions. The neural analysis was performed with respect to facial AUs, focusing on 400–700 ms before and after the start of AU elicitation by the subject monkey.

Neural activity was normalized according to the baseline activity before the relevant block, using the same window length (300 ms) to calculate the mean and SD of the firing rate (FR).

Therefore, the normalized (*z*-scored) FR was calculated as follows:

$${\text{FR}}_{\text{normalized}}=\frac{\text{FR}-{\text{mean}}_{\text{baseline}}}{{\text{SD}}_{\text{baseline}}}.$$

### Data availability

A custom code for automatic MaqFACS recognition and data analysis was written in MATLAB R2017b (https://www.mathworks.com/). The code described in the article is freely available online at https://github.com/annamorozov/autoMaqFACS. The code is available as in Extended Data 1.

10.1523/ENEURO.0117-21.2021.ed1 Extended Data 1 The archive “autoMaqFACS_code.zip” contains MATLAB code for autoMaqFACS classification. Download Extended Data 1, ZIP file

## Results

### Eigenfaces—unraveling the hidden space of facial expressions

**Figure 4.**
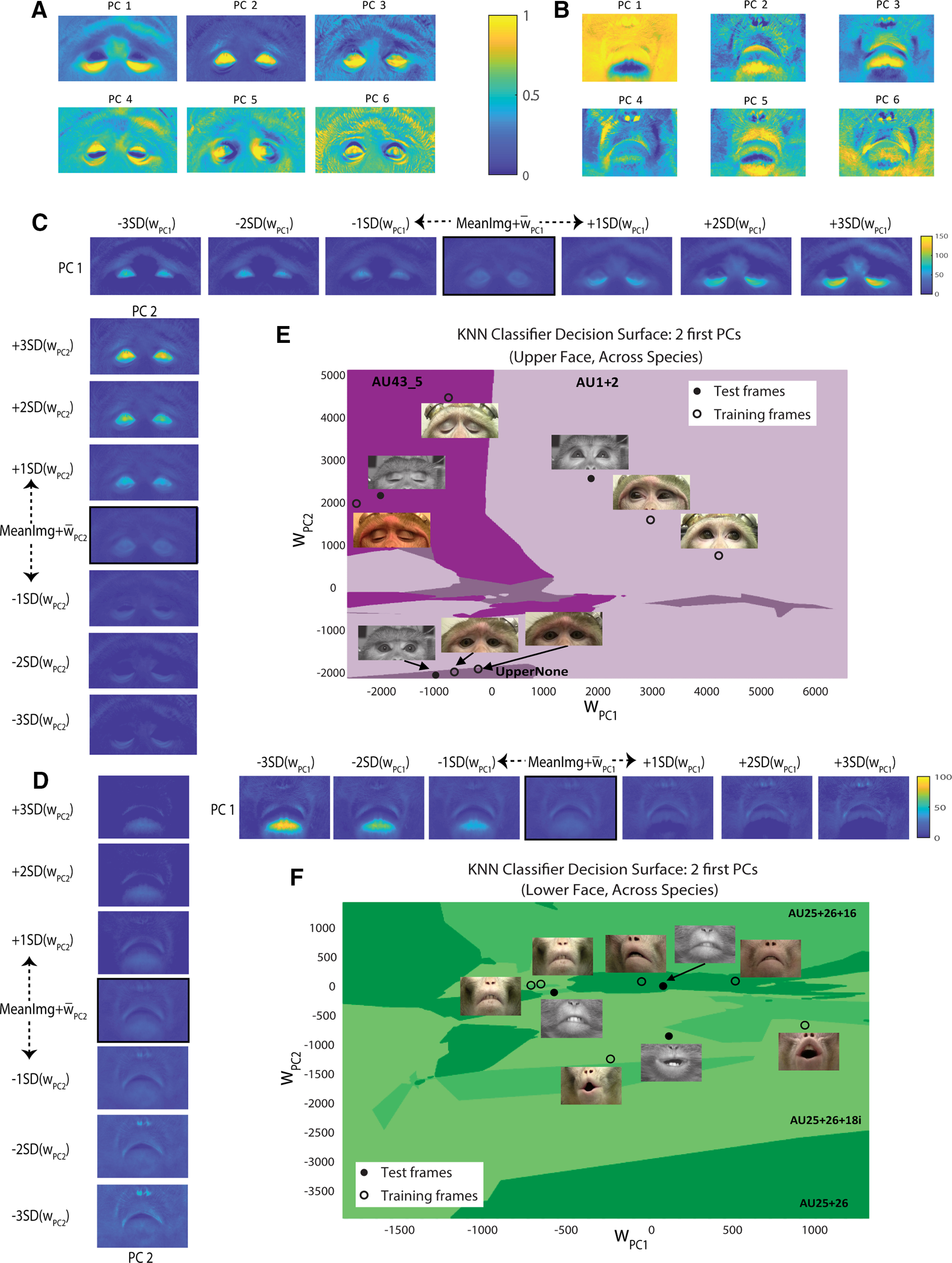
Eigenfaces analysis. ***A***, Example of eigenfaces: six first eigenfaces (PCs) of one of the upper face training sets, containing all five Rhesus subjects from RD. The grayscale values were normalized to the 0–1 range, and the image contrast and color map were adjusted for a better representation. The color bar corresponds to pixel grayscale values. ***B***, Same as ***A***, but for lower face. ***C***, Example of the information coded by the first two eigenfaces. Top, The image sequence demonstrates the first eigenface from ***A***, added to the mean image (MeanImg) and varied. Middle, Mean image of the training set (described in ***A***), with the first eigenface added after being weighted by its mean weight ( ${\bar{w}}_{\text{PC1}}$). In each sequence, the weights were varied from −3 to +3 SDs from the mean weight, and the weighted PC was then added to the mean image of the training set. This procedure resulted in a different facial image for each 1 SD step. The images in the sequence are ordered from left to right: the first image contains the variation by −3 SDs (i.e., PC1 weighted by −3 SDs of its weights and added to the middle image), and the last one is the variation by +3SD. Bottom, Same as top but for the second eigenface (PC2). The image sequence is ordered from bottom to top. The grayscale values were normalized to the 0–150 range, and the image contrast and color map were adjusted for a better representation. The color bar corresponds to pixel grayscale values and is mutual for both the top and bottom schemes. ***D***, Same as ***C***, but for lower face and with grayscale normalization to a range of 0–100. ***E***, Example of decision surface for upper face KNN classifier, trained for generalization across species. The training set is the one described in ***A***, and the test set is Fascicularis monkey D frames from FD. The decision surface is presented along the first two dimensions: weights of PC1 and PC2 ( $w_{\text{PC1}}$ and $w_{\text{PC2}}$, correspondingly). Each colored region denotes one of the three upper face AU classes. The frames in color are training set images, and the grayscale frames are from the test set. The classification decision is based on the proximity of the test frames to samples of a certain class in this compressed subspace. For better illustration, the images shown here are frames after alignment, but before the neutral frame subtraction. ***F***, Same as ***E*** but for the lower face and Fascicularis monkey B from FD test set.

Intuitively, light and dark pixels in the eigenfaces (Fig. 4*A*,*B*) reveal the variation of facial features across the dataset. To further interpret their putative meaning, we varied the eigenface weights to demonstrate their range in the training set, producing an image sequence for each PC (Fig. 4*C*,*D*). This suggests that PC1 of this upper face set (Fig. 4*C*, top, left to right) codes brows raising (AU1 + 2) and eyes opening (AU43_5). In contrast, PC2 resembles eye closure (Fig. 4*C*, bottom, bottom-up). Similarly, PC1 of the lower face set (Fig. 4*D*, top, left to right) probably describes nose and jaw movement. Finally, PC2 for the lower face (Fig. 4*D*, bottom, bottom-up) plausibly corresponds to nose, jaw, and lip movements, reminding the transition from lips pushed forward (AU25 + 26 + 18i) to depressed lower lip (AU25 + 26 + 16).

To illustrate the eigenspace concept, we present decision surfaces of two trained classifiers (Fig. 4*E*,*F*), along their first two dimensions (the weights of PC1 and PC2) that account for changes in facial appearance in Figure 4, *C* and *D*. We show several training and test samples along with their locations following the projection onto the eigenspace. The projection of the samples is performed to estimate their weights, which are then used by the classifier as predictors.

### Parameter selection

**Figure 5.**
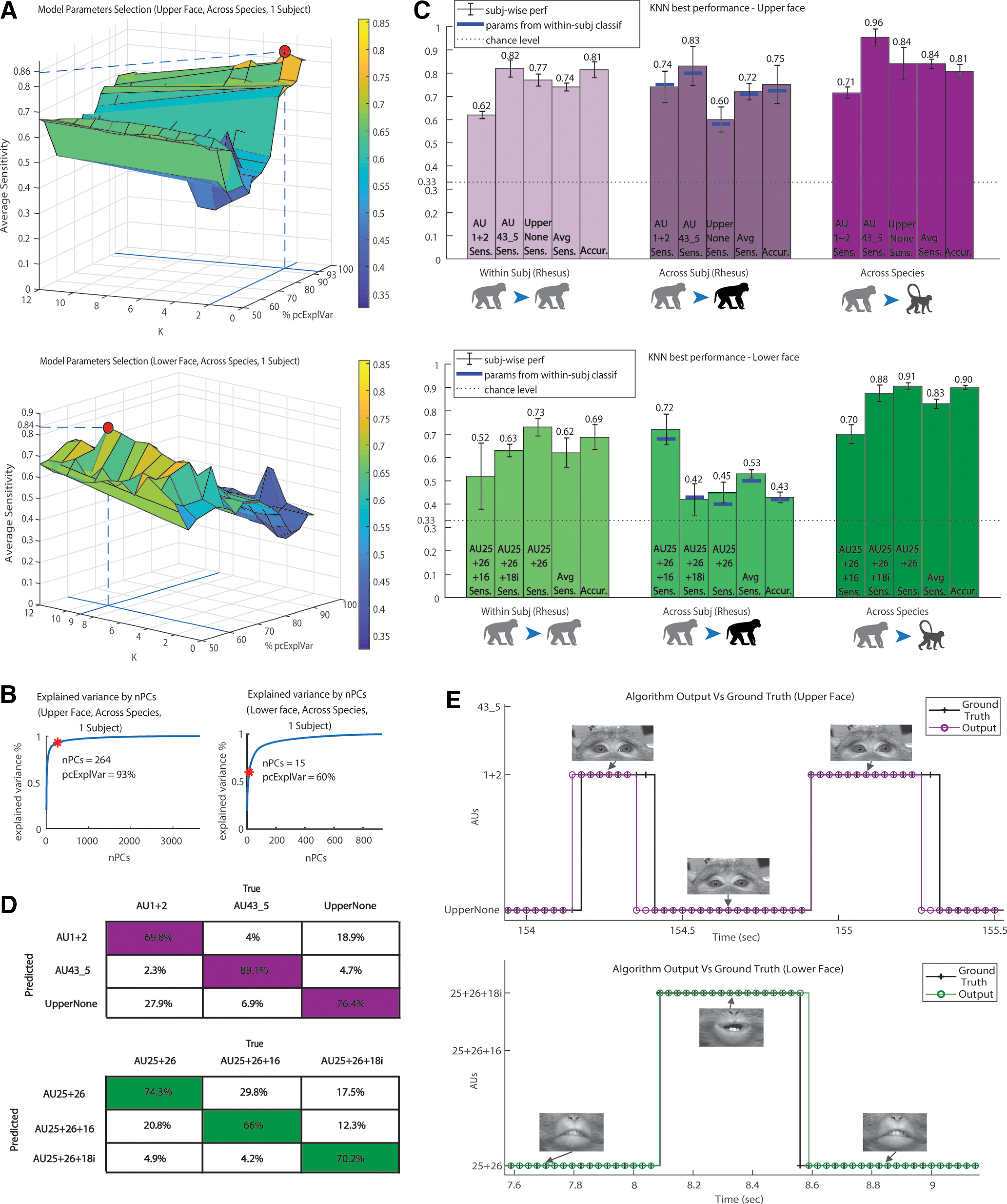
Results for parameters selection and model performance. ***A***, Top, Example of parameter selection for upper face KNN classifier, trained for generalization across species. The training set in the example is the one described in Figure 4*A*, the test set is monkey D frames from FD, and the distance metric is set to be Euclidean. The surface represents the performance of KNN classifiers with two parameters varied: *k* (number of nearest neighbors, varied from 1 to 12), and the percentage of the training set variance explained by the eigenfaces (pcExplVar, varied from 50% to 95%). The *z*-axis is the average sensitivity value of each model (i.e., average of the sensitivity values for the classification of three upper face AUs). The red dot denotes the highest point on the surface and hence the parameters yielding the best performance. With the selected parameters *k* = 2 and pcExplVar = 93%, the model average sensitivity value is 0.86. Bottom, Same as the top but for the lower face. The training set is one of the lower face training sets, containing all five Rhesus subjects from RD, and the test set is monkey D frames from FD. The distance metric is set to be Euclidean. The selected model has the average sensitivity of 0.84 with the following parameters: *k* = 9, pcExplVar = 60%. ***B***, The curves demonstrate the number of eigenfaces that should be used to cumulatively capture a given percentage of the dataset variance. The red asterisk denotes the pcExplVar parameter value selected in ***A***. Left, The curve corresponds to the dataset described in ***A***, top. To express 93% of the dataset variance, at least 264 vectors (eigenfaces) should span the eigenspace. Right, Same as left but regarding ***A***, bottom. To express 60% of the dataset variance, at least 15 vectors (eigenfaces) should span the eigenspace. ***C***, Best performance of KNN classification for each generalization type. Each bar group contains five bars (from left to right), as follows: three bars describing the classifier’s sensitivity for single AUs; sensitivity averaged for three classified AUs; and the total accuracy of the classifier. The mean and the error are calculated regarding the recognition performance on a new subject. The horizontal dashed line denotes the chance level. The first bar group demonstrates the results for generalization of the classification within the same Rhesus subject [within subject (Rhesus): training on videos of a subject and testing on a new video of the same subject]. The second group shows the generalization performance of a classifier to new Rhesus subjects [across subjects (Rhesus): training on videos from several subjects and testing on videos of a new subject]. The blue lines denote the performance of the classifier across subjects using the parameters selected in the within-subject (Rhesus) case. The third group displays the generalization performance to new Fascicularis subjects (across species: training on videos from several Rhesus subjects and testing on videos of a new Fascicularis subject). In this case, the parameters should be tuned for each Fascicularis subject, and the results are the mean performance of two parameter sets (for the two Fascicularis subjects). Top, Performance for upper face. Bottom, Performance for lower face. ***D***, Averaged confusion matrices of the KNN best performance results (of the three cases presented in ***C***). The columns in each matrix represent the true labels, and the rows stand for the predicted labels. Top, Upper face confusion matrices. Bottom, Lower face confusion matrices (Extended Data Fig. 5-1, confusion matrix of inter-rater variability). ***E***, Example of the KNN classification performance demonstrating correctly recognized frames along with some recognition errors. Each data point denotes a frame in a video. The classified AUs (magenta and green lines) are shown in comparison with the ground truth labels (black lines). Video time is displayed in the *x*-axis. Sample frames of the original video stream (after alignment and ROI cropping) are shown above the lines. The video for the example is taken from FD. Top, Output example for upper face video. Bottom, Output example for lower face video.

Example of parameter selection (see Materials and Methods) for a Fascicularis subject is shown in Figure 5*A*. Interestingly, this upper face classification required much larger pcExplVar (93% vs 60% in the lower face; the difference observed in both Fascicularis subjects). Specifically, this upper face classifier achieved its best performance with 264 PCs, opposed to the lower face classifier succeeding with only 15 PCs (Fig. 5*B*). The most likely explanation is the large difference between the training set sizes (upper face, 3639 images; vs lower face, 930 images). Additionally, the eye movement in the upper face images may require many PCs to express its variance.

In contrast, the pcExplVar parameter behaved differently for generalizations within and across Rhesus subjects: their best upper face classifiers required pcExplVar of 85%, and 83% in the lower face sets. The notable difference between the parameters of these datasets suggests that one should tune a different parameter set for each dataset. Generally, the Rhesus dataset required much larger pcExplVar to describe the lower face than the Fascicularis dataset.

### Performance analysis

Overall, the best parameter set for generalization to a new video within subject (Rhesus) using KNN (see Materials and Methods), performed with 81% accuracy and 74% $\bar{TPR}$ per subject for upper face, along with 69% accuracy and 62% $\bar{TPR}$ for lower face, where the chance level is 33% (Fig. 5*C*, left). The best generalization across subjects (Rhesus) yielded $\bar{TPR}$ values of 72% and 53% for upper and lower face, respectively, with corresponding accuracy of 75% and 43% (Fig. 5*C*, middle), compared with 33% chance level. The better performance in the upper face may be explained by its larger number of subjects in the CV (four in the upper face, only three in the lower face) and by greater number of examples available for training. Interestingly, applying the best parameter set of generalization within subject to classifiers generalizing across subjects, produced close-to-best performance (upper face, 71% $\bar{TPR}$; lower face, 50% $\bar{TPR}$). This finding suggests that tuning KNN parameters for generalization within Rhesus subjects, might be enough also for across-Rhesus-subjects generalization.

The finest results, however, were achieved in generalization between species with 84% $\bar{TPR}$ for upper face and 83% for lower face, with corresponding accuracy of 81% and 90%, concerning a 33% chance level (Fig. 5*C*, right). To examine whether our findings depend on the particular classification algorithm, we additionally tested this generalization with a multiclass SVM approach. This improved the $\bar{TPR}$ to 89% for both ROIs, indicating the advantage of using eigenface-based techniques for MaqFACS AUs classification.

Finally, we have also compared the performance of the classifier to the human coders to determine whether the algorithm is superior or inferior to the average, the slow and somewhat subjective human decision. Because of the variability between raters, we found that that the algorithm was more accurate for certain AUs, whereas the human raters were more accurate for other AUs (Extended Data Fig. 5-1, data). Specifically, for UpperNone AU, the classifier had an average sensitivity of 84% versus 81% in the human coding, and for AU 1 + 2 its average sensitivity was 71% versus a raters’ sensitivity of 92.3%. For AU 43_5, the classifier performed with an average sensitivity of 96%, which is similar to the sensitivity of the human coders. For the lower face, the average sensitivity values of the classifier for AU 25 + 26 + 16, AU 25 + 26 + 18i, and AU 25 + 26 were 70%, 88%, and 91% as opposed to the 63.6%, 100%, and 87.5% sensitivity of the human coders, respectively. Overall, our method generalized to Fascicularis monkeys with an average accuracy of 81% for upper face and 90% for lower, compared with the human IRR of 88%.

Altogether, the upper face KNN classifiers (Fig. 5*D*, top) separated AU43_5 well and had typical confusions between UpperNone and AU1 + 2. Most lower face misclassifications (Fig. 5*D*, bottom) were between AU25 + 26 + 16 versus AU25 + 26 and AU25 + 26 + 18i versus AU25 + 26. Characteristic outputs from the system are shown in Figure 5*E*.

### Behavioral analysis

To demonstrate the potential applications of our method, we used it to analyze the facial expressions produced by subject monkeys when exposed to a real-life “intruder” (Fig. 2, Extended Data Fig. 2-1, 2-2, 2-3; Pryluk et al., 2020). The subject monkey was sitting behind a closed shutter, when the intruder monkey was brought into the room (the enter period). The shutter opened, allowing the two monkeys to see each other 18 times. After the last closure of the shutter, the intruder was taken out from the room (exit period).

As the subject monkey was in head immobilization, the facial expressions produced under these conditions were a reduced version of the natural facial expressions that often include head and body movements. To test the ethological validity of such reduced, or schematic, facial expressions, we determined whether they carry signal value (i.e., whether they are sufficient to elicit a situation-appropriate reciprocation for a social partner). We found that when monkeys familiar with each other found themselves in an unusual situation (open shutter), they reassured each other with reciprocal lip-smacking facial expressions, as shown in Extended Data Figures 2-1, 2-2, and 2-3. We verified, therefore, that multiple pairs of monkeys can meaningfully communicate with each other when one of the social partners is in head immobilization.

**Figure 6.**
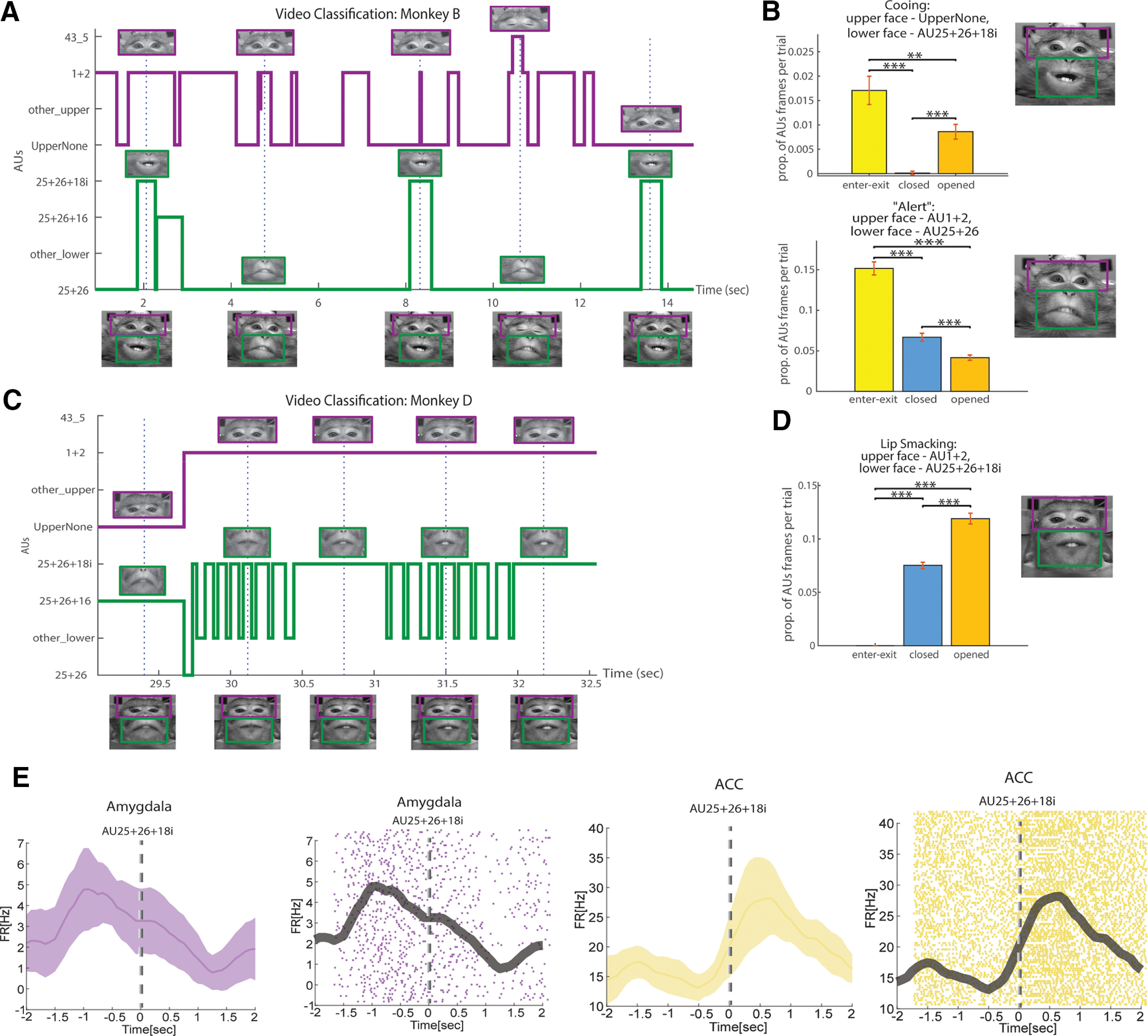
Examples of the method applications. ***A***, Example of the final system output for monkey B from FD. Classification labels are presented on the *y*-axis, while the frame time of the video stream is on the X. “Other_upper” and “other_lower” labels are for video frames that were not part of the task of the classifier but exist in the original video and were labeled manually. Frames of the original video (with no preprocessing) are shown on the bottom, and the dashed lines denote their corresponding timing. The magenta and green lines demonstrate the outputs from the upper face and lower face algorithms, respectively. Images above the output lines exhibit the frames as they were processed in the algorithm, after alignment and ROI cropping. The estimated locations of the ROIs, comprising the full facial expressions, are illustrated in frames on the bottom by magenta and green rectangles (the positions are not precise since the original images on the bottom are not aligned). ***B***, Facial expression analysis following classification of frames. Bars demonstrate the proportion of a specific facial configuration in monkey B (from FD) elicited during one block of the experiment described in Figure 2. This value is calculated as the ratio between frames containing the combination of AUs and the total frames per trial. Yellow bars denote the block part when the intruder monkey enters and exists the room, the blue bar is for phases with the closed shutter (after the first shutter opening and before its last closure), and the orange bars stand for periods of open shutter. An example image of the analyzed expression is shown on the right (taken from the examples in ***B***). Top, Proportions of cooing facial expression events composed of UpperNone AU for the upper face and AU25 + 26 + 18i for the lower face. Bottom, Same as in top, but for “alert” facial expression: upper face, AU1 + 2; lower face, AU25 + 25 (Extended Data Fig. 6-1*a*, analysis following classification by human coders; **p < 1e-2; ***p < 1e-3). ***C***, Same as ***A*** but for monkey D from FD. ***D***, Same as ***B*** but for monkey D from FD and lip-smacking facial expression with upper face AU1 + 2 and lower face AU25+26 + 18i (Extended Data Fig. 6-1*b*, analysis following classification by human coders). ***E***, PSTHs and raster plots of one neuron in the amygdala and one in the ACC, temporally locked to the socially associated AU25 + 26 + 18i, during monkey intruder block.

Statistical analysis of classification results for subject monkey B (Fig. 6*A*) revealed that in the presence of an intruder, he produced several facial expressions including UpperNone and AU25 + 26 + 18i, often associated with cooing behavior. Cooing was more frequent during the enter–exit and open-shutter periods, than during closed-shutter periods (Fig. 6*B*, top, Extended Data Fig. 6-1*a*, left; χ^2^ test, *p* < 1e-3). Moreover, subject B produced an AU1 + 2 and AU25 + 26 combination more frequently during the enter–exit and closed-shutter periods, than during the open-shutter periods (Fig. 6*B*, bottom, Extended Data Fig. 6-1*a*, right; χ^2^ test, *p* < 1e-3). We interpret this pattern as an expression of the alertness and interest of the monkey in events that were signaled by auditory but not visual inputs. Similarly, subject monkey D (Fig. 6*C*) produced AU1 + 2 and AU25 + 26 + 18i together most frequently when the intruder was visible and on occasions when the shutter was closed (intruder behind the shutter), but infrequently during the enter–exit periods (Fig. 6*D*, χ^2^ test, Extended Data Fig. 6-1*b*, χ^2^ test, *p* < 1e-3). In a social context, this pattern is associated with the lip-smacking behavior (Parr et al., 2010), representing an affiliative, appeasing social approach (Hinde and Rowell, 1962).

10.1523/ENEURO.0117-21.2021.f6-1 Figure 6-1 Facial expression analysis from ground truth labeling. Facial expressions analysis following frames classification by a human coder. Same as in Figure 5, *C* and *E*, but deduced from ground-truth labels. ***a***, Monkey B from FD. ***b***, Monkey D from FD. Download Figure 6-1, TIF file.

### Neural analysis

Finally, to validate the concept and strengthen the relevance of automatic MaqFACS for neuroscience applications, we used our method to determine whether neural activity recorded from brain regions involved in facial communication (see Materials and Methods) is related to specific AUs (Fig. 2). Indeed, neurons in the amygdala and ACC were previously shown to respond with changes in firing rate during the production of facial expression (Livneh et al., 2012). In the interaction block of monkeys, responses were computed from the time when the subject monkey started initiating AU25 + 26 + 18i (see Materials and Methods). Reanalyzing the previously obtained data (Pryluk et al., 2020) showed that neurons responded before (Fig. 6*E*, left) or after (Fig. 6*E*, right) the production of the socially meaningful AU25 + 26 + 18i. This finding supports the hypothesis that these regions hold neural representations for the production of single AUs or socially meaningful AU combinations.

## Discussion

This work pioneers the development of an automatic system for the recognition of facial action units in macaque monkeys. We based our approach on well established methods that were successfully applied in human studies of facial action units (Donato et al., 1999). Our system achieved high accuracy and sensitivity, and the results are easily interpretable in the framework of facial communication among macaques. We tested our algorithm using different macaque video datasets in the following three different configurations: within individual Rhesus monkeys; across individual Rhesus monkeys; and across Rhesus and Fascicularis monkeys (generalizing across species). Performance (recognition rates) was obtained for both upper face and lower face using several classification approaches, indicating that the success of this method does not depend on a particular algorithm.

We aimed to build on commonly used and well established tools to enhance applicability and ease of use. The pipeline of our system includes (1) alignment to predefined facial landmarks, (2) definition of upper and lower face ROIs, (3) cropping the images to ROIs, (4) generation of (difference) δ-images, (5) creation of lower and upper face δ-image databases, (6) eigenfaces analysis, and (7) classification. Our classification algorithm uses supervised learning, and its main challenge is the need of a labeled dataset for training. Likewise, to generalize between species, a parameter fine-tuning should be performed on the new species dataset. This requires a sample-labeled set of the new species images. The other manual operations are rather simple and not time consuming. They include a choice of neutral frames and annotation of seven landmark points on a mean neutral image of a video.

Interestingly, unlike the within-Rhesus classifications, the generalization between species required a larger number of components (explained variance) for classification of upper face AUs than for lower face AUs. This might suggest that a separate set of parameters should be fine-tuned for each dataset and ROI (lower and upper face). On the other hand, our findings show that tuning parameters for generalization of within-Rhesus subjects might suffice also for generalization of across-Rhesus subjects. Further, and somewhat surprisingly, the across-species generalization performed better than within-species and across-Rhesus subject generalizations. One possible explanation is that, unlike in the Rhesus dataset, the Fascicularis dataset had better conditions for automatic coding, as its videos were well controlled for angle, scale, illumination, stabilization, and occlusion. This finding has an important implication, as it shows that training on a large natural set of behaviors in less controlled videos (Extended Data Fig. 3-1) can later be used for studying neural substrates of facial expressions in more controlled environments during electrophysiology (Livneh et al., 2012; Pryluk et al., 2020).

A direct comparison to the performance of human AU recognition systems is not straightforward. The systems designed for humans are highly variable because of differences in subjects, validation methods, the number of test samples, and the targeted AUs (Sariyanidi et al., 2015). In addition, some human datasets are posed, possibly exaggerating some AUs, while our macaque datasets are the results of spontaneous behavior. Automatic FACS achieve great accuracy (>90%) in well controlled conditions, where the facial view is strictly frontal and not occluded, the face is well illuminated, and AUs are posed in a controlled manner (for review, see Barrett et al., 2019). When the recordings are less choreographed and the facial expressions are more spontaneous, the performance drops (Benitez-Quiroz et al., 2017, drop to below 83%). Our MaqFACS recognition system performed comparably with the human automated FACS systems despite the spontaneous nature of the macaque expressions and lack of controlled settings for the filming of the Rhesus dataset.

We showed that our method can be used to add detail and depth to the analysis of neural data recorded during real-life social interactions between two macaques. This approach might pave the way toward experimental designs that capture spontaneous behaviors that may be variable across trials rather than rely on perfectly repeatable evoked responses (Krakauer et al., 2017). A departure from paradigms that dedicate less attention to the ongoing brain activity (Pryluk et al., 2019) or internal state patterns (Mitz et al., 2017) will increase our ability to translate experimental finding in macaques to similar finding in humans that target real-life human behavior in health and disease (Adolphs, 2017). Specifically, this will allow internal emotional states and the associated neural activity that gives rise to observable behaviors to be modeled and studied across phylogeny (Anderson and Adolphs, 2014). Indeed, a novel study in mice reported neural correlates of automatically classified emotional facial expressions (Dolensek et al., 2020). Finally, this system could become useful for animal welfare assessment and monitoring (Descovich et al., 2017; Carvalho et al., 2019; Descovich, 2019; for review, see McLennan et al., 2019) and in aiding the 3R framework for the refinement of experimental procedures involving all animals (Russell and Burch, 1959).

Given that macaques are the most commonly used nonhuman primate species in neuroscience, an automated system that is based on facial action units is highly desirable and will effectively complement the facial recognition systems (Loos and Ernst, 2013; Freytag et al., 2016; Crouse et al., 2017; Witham, 2018) that address only the identity of the animal, but not the behavioral state. Compared with the recently introduced method for recognition of facial expressions in Rhesus macaques (Blumrosen et al., 2017), our system does not rely on complete stereotypical and frequent facial expressions; rather, it classifies even partial, incomplete, or ambiguous (mixed) and infrequent facial expressions given by a combination of action units. Although our system requires several manual operations, its main potential lies in automatic annotation of large datasets after tagging an example set and tuning the parameters for the relevant species or individuals. We prototyped our system on six action units in two facial regions (upper and lower face) but more advanced versions are expected to classify additional action unit combinations, spanning multiple regions of interest and tracking action units as temporal events. Further refinement of our work will likely include additional image-processing procedures, such as object tracking and segmentation, image stabilization, artifact removal, and more advanced feature extraction and classification methods. These efforts will be greatly aided by large, labeled datasets, which are emerging (Murphy and Leopold, 2019) to assist ongoing efforts of taking cross-species and translational neuroscience research to the next step.

## Synthesis

Reviewing Editor: Mark Laubach, American University Decisions are customarily a result of the Reviewing Editor and the peer reviewers coming together and discussing their recommendations until a consensus is reached. When revisions are invited, a fact-based synthesis statement explaining their decision and outlining what is needed to prepare a revision will be listed below. The following reviewer(s) agreed to reveal their identity: Jean-René Duhamel, Jessica Taubert.

Both reviewers judged that your manuscript makes a novel and useful contribution to the community. Please address all points raised by them both in your resubmission, and also be sure to address three points that came in the consultation forum for your manuscript.

Reviewer #1:

This manuscript presents a novel approach for the classification of macaque facial expressions, based on MaqFACS, a macaque equivalent of Eckman’s human Facial Action Coding System, developed by Parr and collaborators (2010). Its aim is to automate the recognition of facial poses. This is an interesting and worthwhile endeavour as facial expression labelling with MaqFACS currently needs to be done painstakingly and manually by certified coding experts. The method presented applies PCA and classifiers commonly used in computer vision (NKK and SVM) to image data bases consisting of facial reactions by Rhesus and Fascicularis monkeys filmed in a laboratory setting.

The classification performance for two subregions of the face (eyes and mouth) studied was remarkable both within subject and within and across species. Information extracted from these two regions allowed to reliably identify several species-typical facial action units such as cooing or lip-smacking.

The authors also present data illustrating the usefulness of the technique in the lab, both for the characterization of behavior and the analysis of neuronal tuning.

This paper therefore makes a useful contribution and will be of interest to the research community interested in behavioral and neural mechanisms of primate emotions and social communication.

The introduction and discussion sections are well-constructed and to the point. The study’s design, image preprocessing, analysis methodology and validation procedures appear sound. Given the size of the data sets used, the results presented can be considered reliable.

Here are a few minor remarks.

- The facial expressions highlighted in yellow in Figure 1B were said to be observed in the recordings though not considered as common facial expressions. But it is not entirely clear if expression rarity was an exclusion criterion or whether all frames (blue type and yellow type) were fed to the classifier. Please clarify.

- Manual definition of landmarks. Some clarifications would be welcome here as well. Given that all monkeys were head-fixed, the animal’s head should in principle always be in the same position in the image. Why then was it necessary to place the landmarks on all neutral frames? Also, is the set of landmarks normalized across monkey identity, i.e. does the processing pipeline involve mapping individual facial landmarks to a standard macaque face template?

- In what way is the Rhesus data less-controlled? There are no obvious differences in testing procedure with the Fascicularis. Both species were tested with their head fixed. The data were therefore obtained from full face images, which seem rather optimal conditions.

- Can the authors comment on the performance accuracy of the classifier versus human coders. Is the algorithm doing better (or worse) for different AUs than what could be expected given the interrater variability?

- The discussion could perhaps include a brief bullet-point summary of the processing pipeline, possibly with an estimation of the duration of the manual preprocessing step, which might be a key point when considering the advantage of using this new method over the original MaqFACS.

Reviewer #2:

In this paper the authors have developed an automatic system for classifying combinations of action units in macaque faces. The rationale for building this system is very clear and the authors promise to deliver a product that will be of general interest to the eNeuro community. However, my methodological concerns are centered on the quality (and ecological validity) of the input used to train the classifier. Additionally, I have some concerns about the two demonstrations of utility (there is insufficient detail provided to evaluate this work). I will now list my concerns in the order they occurred to me during my reading.

Behavioral analysis.

Insufficient methodological detail. Could the authors explain the details of the intruder task, including timing parameters and recording materials. How many trials were run on Monkeys B and D?). Were images of monkeys B and/or D used to train the classifier?

What was the ground truth? The statistical results provided indicate that the classifier tagged the behaviors of Monkey D and D with labels, but there is no indication of whether these labels were accurate. Could the behaviors of Monkey B and D during the intruder experiment be coded by a qualified MacFACS expert to verify the performance of the classifier?

¬¬¬

Neural analysis.

Insufficient methodological detail. From my reading it is difficult to understand what analysis was performed here and how these results validate the classifier.

In the materials and methods section is says that that the subjects were filmed while seated and head fixed but this does not sound very natural. What events or tools were used to elicit different expressions from the subjects? Was there any attempt to verify that these are recognizable facial expressions to rhesus monkeys?

From the consultation session:

The following issues were raised in the consultation session between me and the reviewers. Please be especially sure to address these points, and document your revisions to address them in your rebuttal. Of particular note is sharing your code before your resubmit the manuscript. You can share the actual address with me in the cover letter.

-Behavioral and neural validation of the method needs more details, as supplementary information if necessary.

-Whether facial expressions generated during head immobilization constitute ecologically meaningful stimuli should be addressed with data or discussed as a potential limitation.

-The software should be shared before the final decision is made on the manuscript.

## Author Response

Response to reviewers:

We thank the reviewers for their constructive comments and suggestions. To address the concerns, we have introduced new data and figures, added new analyses and revised the manuscript accordingly.

We first address the three points that came in the consultation forum, and then elaborate more in the answers for each individual reviewer. Please find below the concerns of the reviewers in italics and our responses in blue.

General:

Behavioral and neural validation of the method needs more details, as supplementary information if necessary

We have added a new Extended Fig. 6-1 to show a better validation of the behavioral analysis. Also, we have added to the Materials and Methods section a more detailed explanation regarding the neural analyses we have performed. Please find additional details in our answer to Reviewer #2.

Whether facial expressions generated during head immobilization constitute ecologically meaningful stimuli should be addressed with data or discussed as a potential limitation.

In response to this important question, we performed new analyses on new data. We documented that the intruder monkey responds to the subject monkey with a socially appropriate facial expression at delays expected during the exchange of facial expressions. We interpreted this behavior to mean, that despite head immobilization the social signals exchanged between the monkeys are recognized by both partners. We illustrate in Extended Fig. 2-1, 2-2 and 2-3 that such behavior indeed occurs systematically. Please find additional details in our answer to Reviewer #2.

The software should be shared before the final decision is made on the manuscript.

We are happy to share the code of our system and the algorithm. It is now published in the open-source repository [URL redacted for double-blind review]. The code is also available as Extended Data.

Reviewer 1:

The facial expressions highlighted in yellow in Figure 1B were said to be observed in the recordings though not considered as common facial expressions. But it is not entirely clear if expression rarity was an exclusion criterion or whether all frames (blue type and yellow type) were fed to the classifier. Please clarify.

We agree that this requires clarification. We now explain in the text that less common facial expressions were used for the classification, but only if these expressions contained relevant action units (i.e. AU’s that were classified). We relied on the premise that all facial expressions - the common ones and the rare ones - can be objectively expressed by a combination of AUs that are well defined and verified in the standardized FACS systems.

Manual definition of landmarks. Some clarifications would be welcome here as well. Given that all monkeys were head-fixed, the animal’s head should in principle always be in the same position in the image. Why then was it necessary to place the landmarks on all neutral frames? Also, is the set of landmarks normalized across monkey identity, i.e. does the processing pipeline involve mapping individual facial landmarks to a standard macaque face template?

We thank the reviewer for pointing out the need for additional clarification. We added a new figure (Extended Fig. 3-1) to illustrate the need for landmarks in each video. This was necessary because unlike in the Fasicularis dataset, the Rhesus dataset required alignment due to minor movements of the camera between frames. Moreover, the camera was placed in a slightly different position for each movie (angle, distance, and height). To address this, we aligned the videos based on seven landmark points on a mean neutral image and found their affine transformation onto predefined “absolute” landmarks. All the videos of all the monkeys were aligned to these predefined landmarks. Notice this approach also has a benefit, as it shows our approach can be generalized to imperfections in the camera/head position, as in many lab implementations. The extended material we added now describes the approach in greater details.

In what way is the Rhesus data less-controlled? There are no obvious differences in testing procedure with the Fascicularis. Both species were tested with their head fixed. The data were therefore obtained from full face images, which seem rather optimal conditions.

As pointed out above, the new Extended Fig. 3-1 and explanations in the text highlight more clearly the differences between the two datasets. Even though for the Fascicularis dataset the camera was installed to be fixed and stable for all sessions, small changes in the subject’s head position were still possible. Overall, we believe this strengthen our findings, showing that it can overcome common setup-related noise.

Can the authors comment on the performance accuracy of the classifier versus human coders. Is the algorithm doing better (or worse) for different AUs than what could be expected given the interrater variability?

This is an important question and touches upon the very essence of why automation is feasible. We have added extended material (see Extended Table 5-1) that aids the comparison of these two approaches. We added a confusion matrix of the raters’ coding for a Fascicularis monkey video. Given some inter-rater variability, the classifier performed slightly better or slightly worse that the human raters. For example, for the lower face, the

average sensitivity of the classifier for AU 25+26+16, AU 25+26+18i and AU 25+26 was

70%, 88% 91% as opposed to 63.6%, 100% and 87.5% sensitivity of the human coders, respectively.

Overall, our method generalized to Fascicularis monkeys with average accuracy of 81%

for upper face and 90% for lower, as compared to the human inter-rater reliability (IRR) of

88%.

We have compared our results to the early results of the automated systems for human FACS recognition in the literature. Bartlett et al., 1996, classified six upper face actions applying eigenfaces analysis on δ-images (difference images) and achieved accuracy of

88.6% on a database of posed facial expressions. In a similar study, Bartlett et al., 2000, obtained accuracy of 79.3% also using δ-images eigenfaces of posed facial expressions database, recognizing 12 AUs (6 upper and 6 lower face AUs). Draper et al., 2003, reported a result of 85% average recognition rate using eigenfaces on posed facial expression dataset, classifying 7 upper face AUs. As expected, the results of such systems for spontaneous movements are poorer. Cohn et al., 2004, attempted to

recognize AU 1+2 (brow raiser), AU 4 (brow lowerer) and AU 0 (no action) in spontaneous

facial behavior which includes also moderate out-of-plane head motion. They reported recognition rate for two-state classification (AU 1+2 versus AU 4) of 89% and recognition rate of 76% for three-state recognition. The conditions of this experiment resemble closely our experiment and our sensitivity measures are also similar.

The discussion could perhaps include a brief bullet-point summary of the processing pipeline, possibly with an estimation of the duration of the manual preprocessing step, which might be a key point when considering the advantage of using this new method over the original MaqFACS

Thank you for this suggestion. We have added a summary of the algorithm pipeline along with a discussion of the manual preprocessing steps.

Reviewer 2:

Behavioral analysis.

Insufficient methodological detail. Could the authors explain the details of the intruder task, including timing parameters and recording materials. How many trials were run on Monkeys B and D?). Were images of monkeys B and/or D used to train the classifier?

We thank the reviewer for this request. Indeed, our description needed more detail. We describe the intruder task in the manuscript including how the monkey intruder was implemented. We also added a figure (Extended Fig. 2-1, 2-2, and 2-3) that illustrates the procedure.

Briefly, single experimental block includes 18 interactions (3 repetitions of 6 trials) with a monkey intruder that is seated behind a fast LCD shutter (<1ms response time, 307mm x

407 mm). When the shutter opens, the monkeys are able to see each other. Each trial is of

9 sec and the shutter is closed for 1 sec between the trials. Under these conditions, we recorded the facial expressions of the subject monkey, along with monitoring the behavior of the intruder. Otherwise, the shutter prevented the monkeys from seeing each other.

The images of monkey B and D were not used to build the eigenspace, but were included in the testing and the validation process of the classifier for tuning its parameters (see Materials and Methods - Validation and model evaluation).

What was the ground truth? The statistical results provided indicate that the classifier tagged the behaviors of Monkey D and D with labels, but there is no indication of whether these labels were accurate. Could the behaviors of Monkey B and D during the intruder experiment be coded by a qualified MacFACS expert to verify the performance of the classifier?

We agree that reporting the behavioral results coded by MaqFACS human rater will provide a more complete understanding of our data. We now report the requested results in Extended Fig. 6-1. This serves as proof of concept for our method with behavioral data. It illustrates the similarities between human expert coding and the automatic classification. In agreement with the automatic classification results, the coding of the rater revealed that monkey B performed cooing behavior during the “enter-exit” and open shutter phases more frequently, than during the closed-shutter periods. Likewise, both the automatic and the human coding show that monkey B had the “alert” expression with higher prevalence during the “enter-exit” and closed-shutter periods, than during the open-shutter ones. The relations are consistent in the classification of Monkey D facial expressions, as well. Both the human and the automatic classifications show that monkey D performed lip-smacking mostly when the intruder monkey was visible, and on occasions when the shutter was closed with the intruder monkey behind it, but not during the “enter-exit” phases, when the intruder was brought or taken out of the room.

Neural analysis.

Insufficient methodological detail. From my reading it is difficult to understand what analysis was performed here and how these results validate the classifier.

Thanks. We have added a more detailed description of the analyses to the Materials and Methods section. Our goal was to introduce a prototype of an automatic method for MaqFACS classification, and to illustrate how it may be used in the neuroscience community. In figure 6 we demonstrate examples of utilizing the automatic algorithm for purposes such as description of facial movement progression (Fig.6A,C), revealing patterns of facial behavior in different conditions (Fig.6B,D) and using the automatic labeling to search for neural responses that are locked to certain facial behaviors (Fig.6E).

In the materials and methods section is says that that the subjects were filmed while seated and head fixed but this does not sound very natural. What events or tools were used to elicit different expressions from the subjects? Was there any attempt to verify that these are recognizable facial expressions to rhesus monkeys?

We agree that the ecological validity of the facial expressions in head-fixed monkeys is an important topic to discuss and to be addressed in future studies (currently ongoing in few labs). Many previous studies from our Labs and others have demonstrated that head-fixed monkeys make distinguishable and context-appropriate facial expressions and in response to the presence of another monkey. This strongly argues for the communicative value of these expressions. We acknowledge that in head-fixed monkeys, a natural open-mouth

threat display, is likely a reduced version of the full display (likely because this expression is often associated with head bobbing). The same facial expressions produced by head- free monkeys may also be a reduced version of the natural expression because a full threat is often associated with lunging forward, crook tail, and a short “stomping” with the forelegs. The important point and observation we used here is that a reduced facial expression, even though a caricature of the full “natural” display, contains the building- blocks for the ethological signal, and hence its value. The evidence for this comes from observation that the social partner responds with socially appropriate reciprocal displays.

To show this in the context of our own dataset, we added Extended Fig. 2-1, 2-2 and 2-3 to illustrate this point. In this figure we show that monkey D (head-fixed), produces a lip- smacking expression towards the intruder monkey. All three intruders (B, P and N) are familiar with monkey D and indeed all three reciprocated the lip-smacking expression of monkey D. We also describe in the manuscript that the facial movements of the Rhesus monkeys were provoked by exposure to a mirror or to videos of other monkeys. Also,

strong evidence from our previous work shows that monkeys produce socially appropriate facial expressions and gaze-mediated interactions toward videos or mirrors (Mosher et al.,

2011, Ballesta et al., 2016, Putnam et al., 2016), and towards real-life intruders (Livneh et al., 2012)

Draper, B.A., et al., Recognizing faces with PCA and ICA. Computer Vision and Image

Understanding, 2003. 91(1-2): p. 115-137.

Bartlett, M.S., et al. Classifying facial action. in Advances in neural information processing systems.

1996.

Bartlett, M.S., et al. Image representations for facial expression coding. in Advances in neural information processing systems. 2000.

Cohn, J.F., et al. Automatic analysis and recognition of brow actions and head motion in spontaneous facial behavior. in 2004 IEEE International

Mosher, C.M., Zimmerman, P.E., Gothard, K.M. (2011) Videos of conspecifics elicit interactive looking patterns and facial expressions in monkeys. Behavioral Neuroscience 25:639-652.

Ballesta, S., Mosher, C.P., Szep, J., Fischl, K.D., Gothard, K.M. Social determinants of spontaneous eye blinks in adult male macaques. Scientific Reports 6;6:38686. doi: 10.1038/srep38686. PMID:27922101

Putnam, P.T., Roman, J.M., Zimmerman, P.E., Gothard KM. (2016) Oxytocin Enhances Gaze- following induced by videos of natural social behavior. Psychoneuroendocrinology. 72:47-53

Livneh, U., Resnik J., Shohat S., Paz R. (2012) Self-monitoring of social facial expressions in the primate amygdala and cingulate cortex. Proc Natl Acad Sci U S A.
