# Supplementary figures and images for: Automatic Recognition of Macaque Facial Expressions for Detection of Affective States
Source: eNeuro. 2021 Dec 9;8(6):ENEURO.0117-21.2021. doi: 10.1523/ENEURO.0117-21.2021 (PMC8664380; doi:10.1523/ENEURO.0117-21.2021)

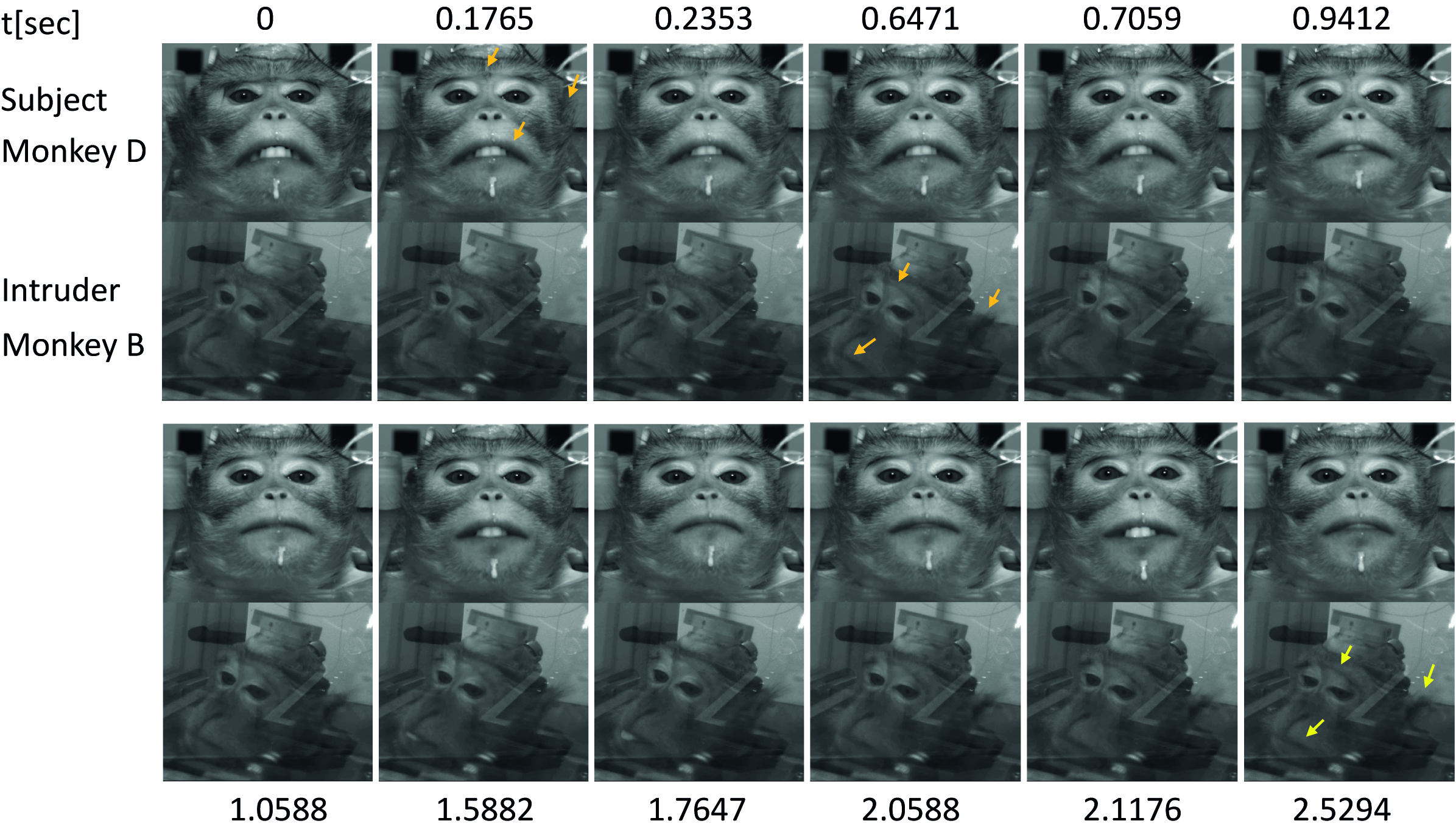

Supplement: Figure 2-1 — Lip-smacking interactions. Examples of dynamics and progression of lip-smacking interactions captured during the monkey–intruder experiment, where the subject monkey is the first to initiate the movement. Each sequence demonstrates sample frames of the Fascicularis subject D with his head fixed (first row), along with the corresponding frames of the intruder Fascicularis monkey (second row). The subject monkey D was filmed using the facial camera (see Materials and Methods). The intruder monkey was filmed using another monitoring camera, from the direction of the subject monkey and through the opened shutter (hence, the reflections on the screen). The time presented relative to the first frame in the sequence, which starts with a neutral expression of the subject monkey. Yellow arrows indicate the change in the movement of brows, ears, and lips at the onset of the lip-smacking movement (for the subject and the intruder monkeys) and the offset of the movement (for the intruder monkey). In the example, sequence with intruder monkey B. Download Figure 2-1, TIF file. [file enu-eN-MNT-0117-21-s02.tif]

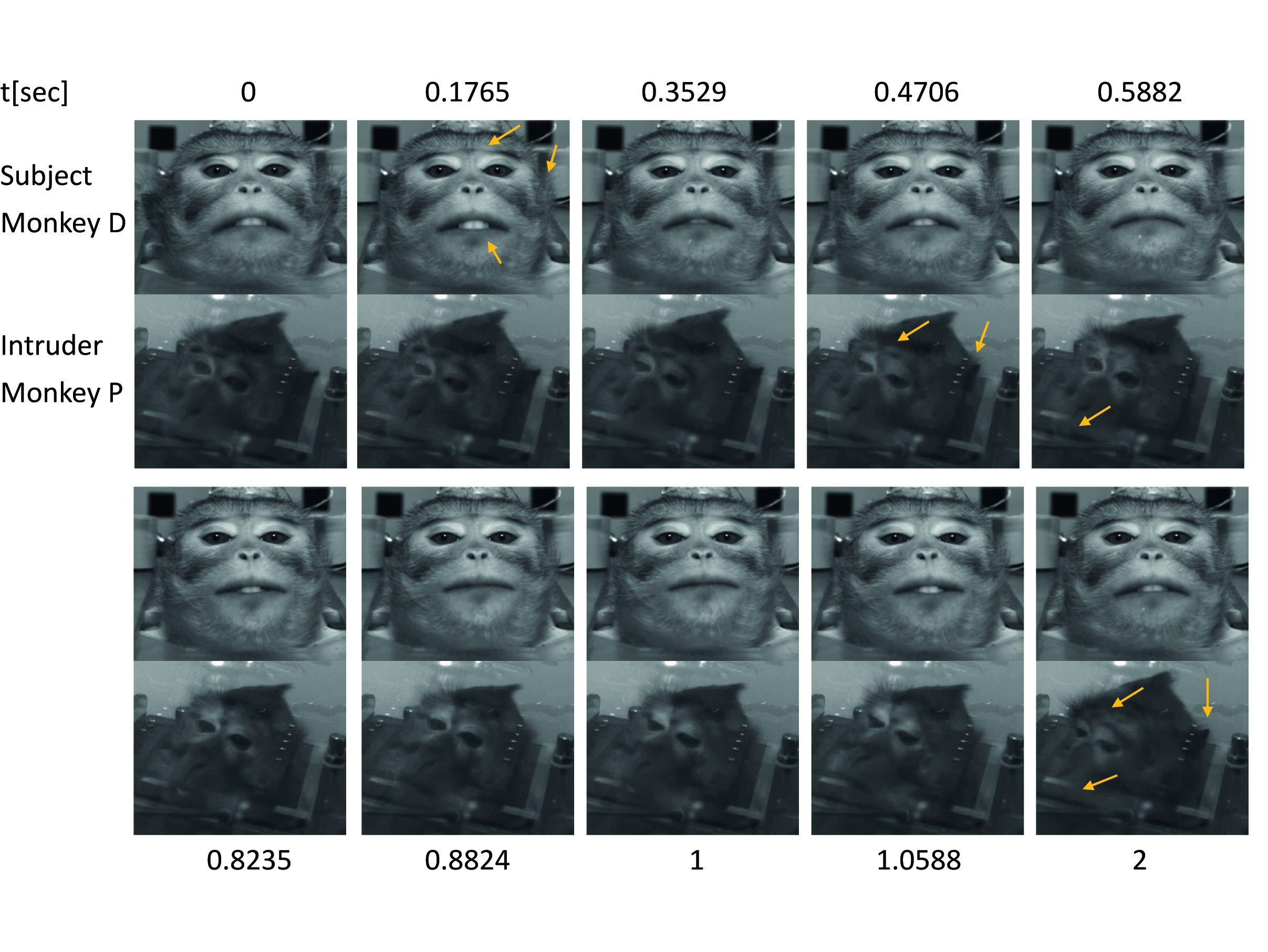

Supplement: Figure 2-2 — Lip-smacking interactions. Same setup as in Figure 2-1, but with intruder monkey P. Download Figure 2-2, TIF file. [file enu-eN-MNT-0117-21-s03.tif]

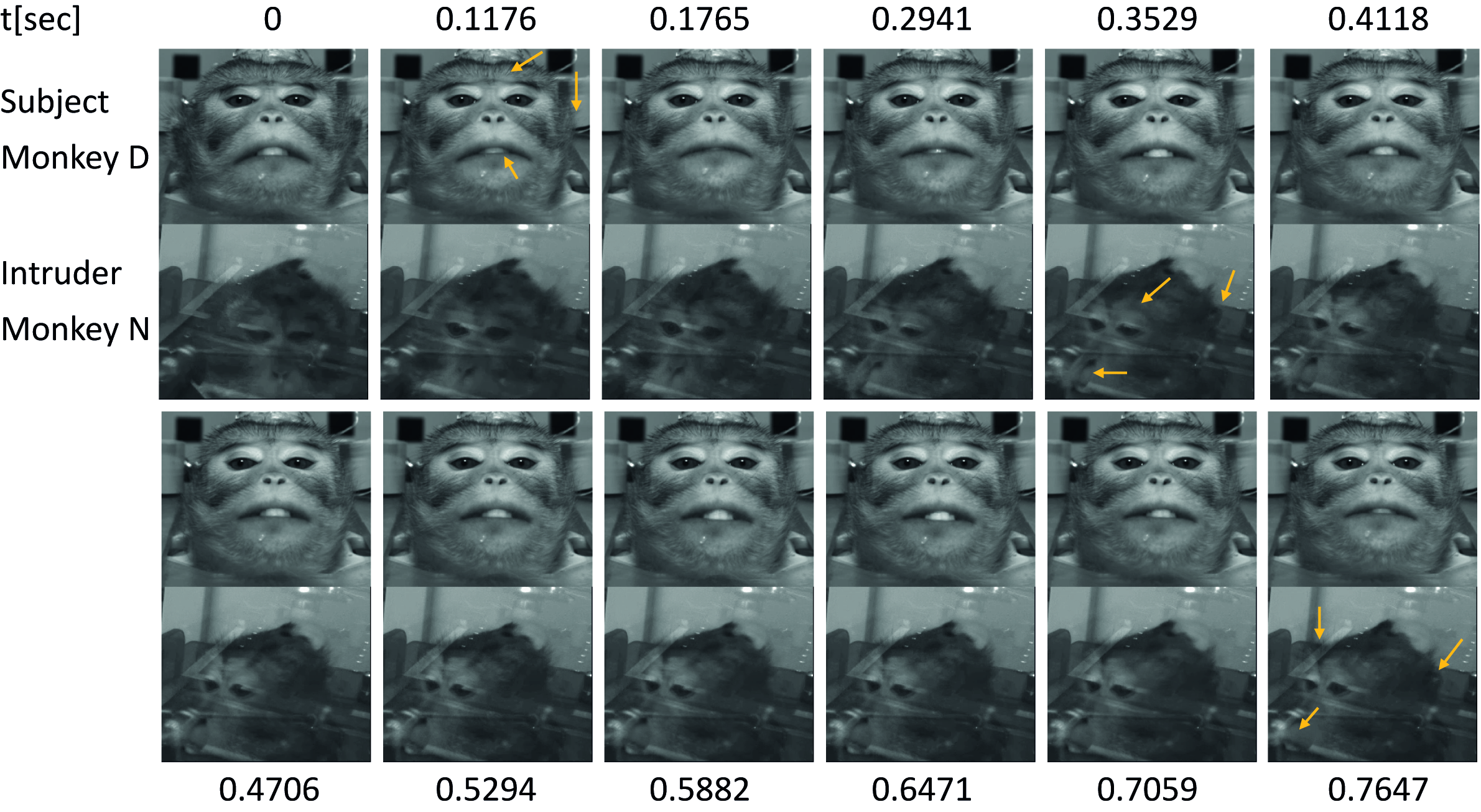

Supplement: Figure 2-3 — Lip-smacking interactions. Same setup as in Figure 2-1, but with intruder monkey N. Download Figure 2-3, TIF file. [file enu-eN-MNT-0117-21-s04.tif]

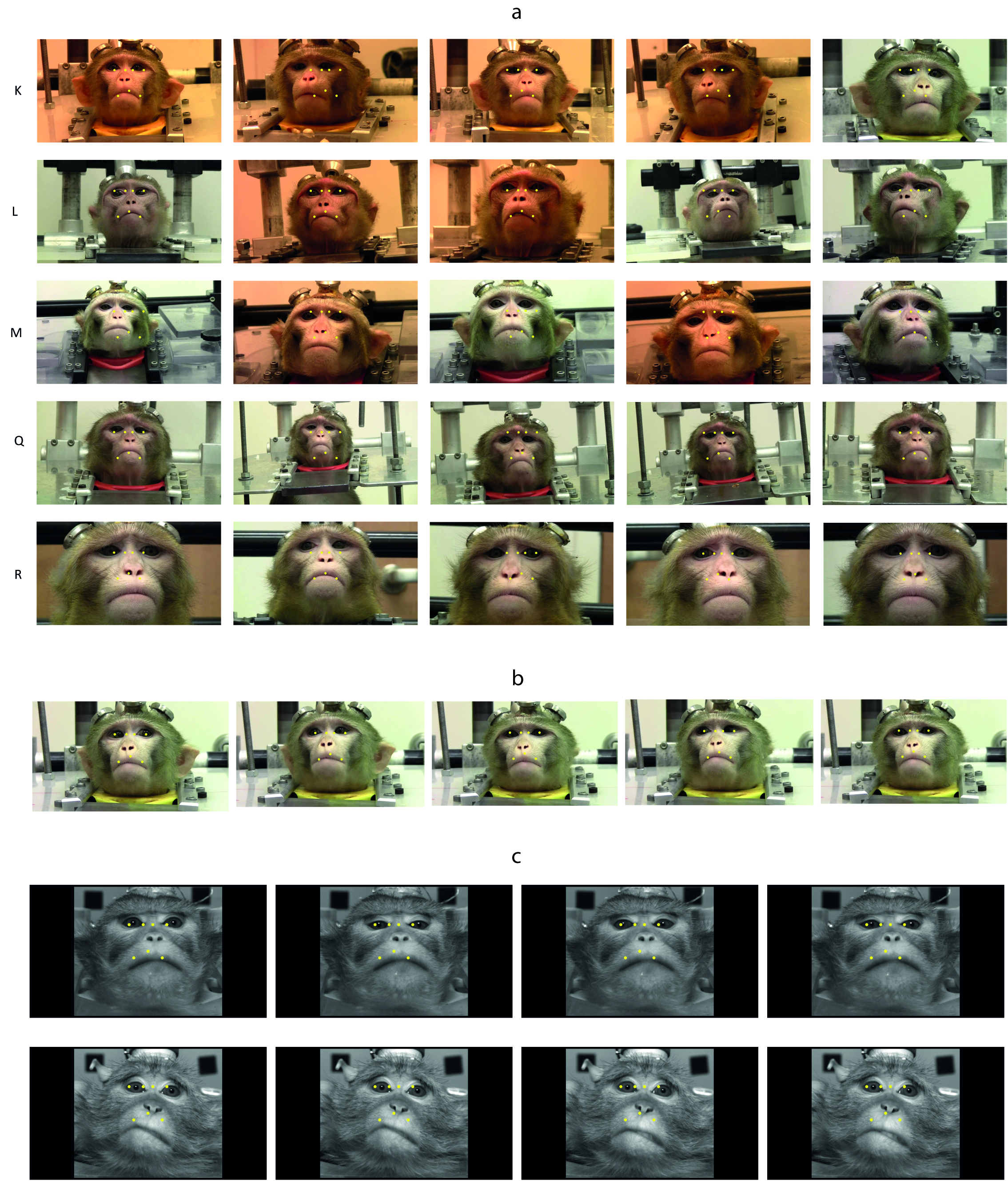

Supplement: Figure 3-1 — Motivation for alignment. Seven reference landmark points (yellow, predefined and common for all videos) displayed on sample neutral frames of original video streams. A, Sample neutral frames from five different videos of each of the five Rhesus monkeys (K, L, M, Q, R). B, Sample neutral frames from one video of Rhesus monkey K. C, Sample neutral frames of the two Fascicularis monkeys (D and B). Download Figure 3-1, TIF file. [file enu-eN-MNT-0117-21-s05.tif]

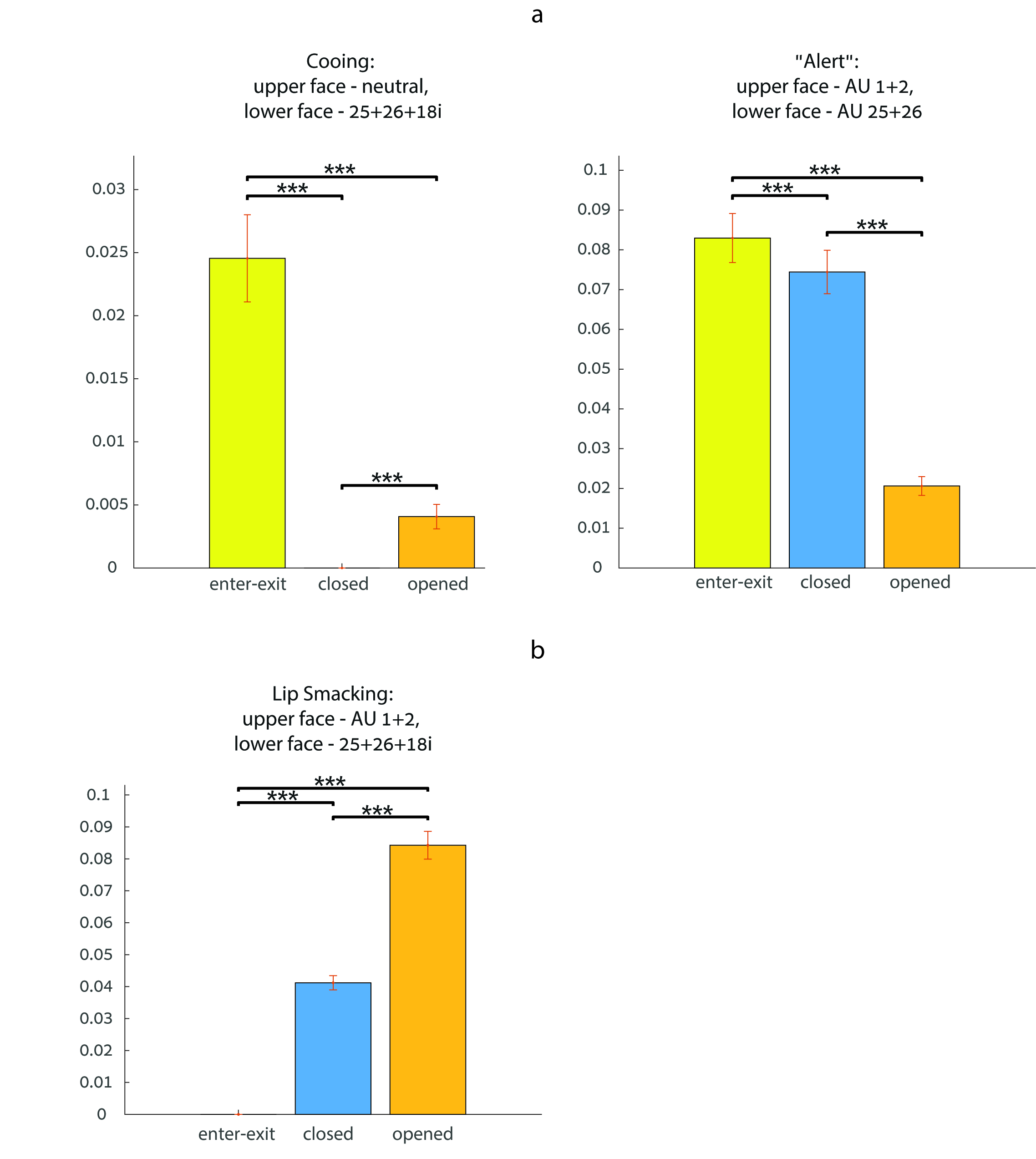

Supplement: Figure 6-1 — Facial expression analysis from ground truth labeling. Facial expressions analysis following frames classification by a human coder. Same as in Figure 5, C and E, but deduced from ground-truth labels. a, Monkey B from FD. b, Monkey D from FD. Download Figure 6-1, TIF file. [file enu-eN-MNT-0117-21-s07.tif]
