# Supplementary material for: Automatic Recognition of Macaque Facial Expressions for Detection of Affective States
Source: eNeuro. 2021 Dec 9;8(6):ENEURO.0117-21.2021. doi: 10.1523/ENEURO.0117-21.2021 (PMC8664380; doi:10.1523/ENEURO.0117-21.2021)
Supplement: Figure 5-1 — Confusion matrix: inter-rater variability. Confusion matrix for the inter-rater variability between two experienced human coders, for a video from FD. “Other Upper” and “Other Lower” represent all the upper face and lower face labels that were not part of the task of the automatic classifier. Download Figure 5-1, DOCX file. [file enu-eN-MNT-0117-21-s06.docx]

**Figure 5-1: Confusion matrix: interrater variability**

|  | AU43_5 | UpperNone | AU1+2 | AU1+2+43_5 | Other  Upper | AU25+26 | AU25+26+18i | Other  Lower | AU25+26+16 |
| --- | --- | --- | --- | --- | --- | --- | --- | --- | --- |
| AU43_5 | 96% | <1% | 0 | 0 | 0 | 0 | 0 | 0 | 0 |
| Upper  None | <1% | 81% | 6.2% | 0 | 6.1% | 0 | 0 | 0 | 0 |
| AU1+2 | 3% | 18.9% | 92.3% | 12.5% | 6.1% | 0 | 0 | 0 | 0 |
| AU1+2+43_5 | <1% | 0 | <1% | 87.5% | 0 | 0 | 0 | 0 | 0 |
| Other  Upper | 0 | <1% | 1% | 0 | 87.8% | 0 | 0 | 0 | 0 |
| AU25+26 | 0 | 0 | 0 | 0 | 0 | 87.5% | 0 | 3.1% | 2.6% |
| AU25+26+18i | 0 | 0 | 0 | 0 | 0 | 1.4% | 100% | <1% | 0 |
| Other  Lower | 0 | 0 | 0 | 0 | 0 | 10% | 0 | 95.5% | 33.8% |
| AU25+26+16 | 0 | 0 | 0 | 0 | 0 | 1.1% | 0 | 1.3% | 63.6% |

Confusion matrix for the interrater variability between two experienced human coders, for a video from FD. “Other Upper” and “Other Lower” represent all the upper-face and lower-face labels which were not part of the task of the automatic classifier.
